# Alternate and Emerging Anticoagulation Strategies for Extracorporeal Membrane Oxygenation: A Scoping Review

**DOI:** 10.3390/jcm15062337

**Published:** 2026-03-18

**Authors:** Akshay Kumar, Nicole Carlo, Rithish Nimmagadda, Juber Dastagir Shaikh, Sourabh Khatri, Vivek Varghese

**Affiliations:** 1 Department of Cardiothoracic Surgery, New York University Langone Health, New York, NY 10016, USA; 2 John Hopkins School of Public Health, Baltimore, MD 21205, USA; ncarlo1@jh.edu; 3 Department of Internal Medicine, One Brooklyn Health, New York, NY 11213, USA; rithishnvs@gmail.com; 4 Department of Neurology, Henry Ford Hospital, Detroit, MI 48202, USA; jubershaikh703@yahoo.com; 5 Independent Health System, Greensburg, PA 15601, USA; sourabhk@gmail.com; 6 Department of Medicine, Prisma Health, University of South Carolina School of Medicine, Columbia, SC 29203, USA

**Keywords:** extracorporeal membrane oxygen, anticoagulation, heparin, bivalirudin, thromboelastometry

## Abstract

**Background**: Unfractionated heparin (UFH) remains the standard anticoagulant for extracorporeal membrane oxygenation (ECMO), despite complications, such as heparin resistance, heparin-induced thrombocytopenia, bleeding and variable pharmacokinetics. This has prompted the search for alternative and novel anticoagulation strategies, including pharmacologic agents, circuit modifications, and monitoring approaches. This scoping review aimed to map the breadth and characteristics of evidence on ECMO anticoagulation strategies beyond UFH. **Methods**: A comprehensive search of peer-reviewed and gray literature was conducted across PubMed, Cochrane, Clinical Trials, WHO Trials Registry, and conference abstracts through manual searches in key journals. Clinical, pre-clinical, and gray literature studies evaluating pharmacologic agents, anticoagulation-free or heparin-sparing, biocompatible circuits, and monitoring innovations were included. Data were charted and synthesized descriptively to identify trends, gaps, and emerging directions. **Results**: A total of 269 records were included. Evidence was highly heterogeneous among study designs, populations, ECMO modalities, and outcome definitions. Most clinical studies were retrospective cohorts and adult-centered, with limited multicenter randomized controlled trials and underrepresentation of neonatal and pediatric populations. Direct thrombin inhibitors were frequently studied and clinically implemented alternatives to UFH. Other agents, including nafamostat mesylate, prostaglandin E1, and factor pathway inhibitors remain early in clinical investigation. Anticoagulation-free strategies and biocompatible circuit technologies were mostly supported through pre-clinical and single-center studies. Monitoring and modeling innovations, like TEG, ROTEM, real-time imaging, and machine learning, are quickly emerging. **Conclusions**: ECMO anticoagulation is transitioning from UFH reliance toward diversified and personalized strategies. Future research should prioritize multicenter randomized controlled trials, standardize protocols, expand to neonatal and pediatric investigation, and integrate strategies.

## 1. Introduction

Extracorporeal membrane oxygenation (ECMO) supports critically ill patients with severe cardiac or respiratory failure, sudden cardiac arrest, acute respiratory distress syndrome (ARDS), or COVID-19 unresponsive to standard therapy [1]. The two main ECMO configurations are venoarterial (VA-ECMO), which supports cardiac and respiratory functions, and venovenous (VV-ECMO), which only provides respiratory assistance [1]. In ECMO, blood is circulated through an extracorporeal circuit, where exposure to a large, foreign biomaterial surface activates the coagulation pathways, the complement system, platelets, and von Willebrand factor, creating a prothrombotic state [2].

A recent ELSO registry analysis reported hemorrhagic complications in 37% of ECMO patients, with 21% experiencing both bleeding and thrombotic events [2]. In a separate report, oxygenator-related thrombosis has been reported in 10–15% of ECMO patients [2]. Together, these findings highlight the need for effective anticoagulation management to maintain hemostatic balance. Unfractionated heparin (UFH) remains the preferred anticoagulant because of its rapid onset, reversibility, and wide availability [3]. However, UFH use is limited by increased bleeding risk, heparin resistance, heparin-induced thrombocytopenia (HIT), and unpredictable pharmacokinetics [1,3]. These limitations have prompted the search for alternative anticoagulation strategies and optimized monitoring approaches.

The literature on alternative and emerging anticoagulation strategies in ECMO is highly heterogeneous and rapidly evolving. Evidence spans diverse patient populations, ECMO configurations, anticoagulation targets, study designs, and outcome definitions. Most studies are single-center, retrospective cohorts, and there is limited high-quality randomized controlled trial data. This heterogeneity limits comparative effectiveness synthesis but highlights potential innovation. Several narrative and systematic reviews have examined ECMO anticoagulation, primarily focusing on UFH management or selected pharmacologic alternatives such as direct thrombin inhibitors and nafamostat mesylate [4,5,6,7,8,9].

While these reviews provide important insights into monitoring strategies, bleeding and thrombotic risks, and comparative effectiveness, they are limited to narrow pharmacologic comparisons or specific populations. Thus, the full scope of emerging approaches is not captured. In contrast, this scoping review maps and synthesizes evidence across pharmacologic agents, anticoagulation-free strategies, circuit modifications, and emerging monitoring and modeling technologies. By including the gray literature, the review’s purpose is to identify emerging trends, innovations, and gaps in ECMO anticoagulation, and identify areas sufficiently developed to support future systematic reviews and comparative effectiveness research.

### Study Objectives and Aims

**Objectives**

**Primary objective:** To map the existing literature on alternative and emerging anticoagulation strategies in ECMO.

**Secondary objective:** To identify strategies, patient populations, and outcomes sufficiently studied to support future comparative effectiveness research and systematic reviews.

**Aims**: The review aims to do the following:

1. Identify the types of alternative and emerging anticoagulation strategies reported in ECMO patients.
2. Describe key characteristics of studies and populations, including the following:
  - Study design (e.g., single-center **vs.** multicenter, geographic location, and clinical setting).
  - Patient population (e.g., adult, pediatric, or neonatal).
  - ECMO modality (VV, VA, or hybrid).
  - ECMO indication (e.g., cardiac failure, ARDS, and COVID-19).
3. Characterize the outcomes assessed, including bleeding, thrombotic events, circuit-related complications, and mortality.
4. Identify trends and evidence gaps to inform future research directions.

## 2. Methods

### 2.1. Study Design

This scoping review was developed from a prior narrative review; therefore, a formal protocol was not registered a priori before data extraction commenced. To enhance transparency, a detailed protocol was retrospectively registered on the Open Science Framework (OSF) on 20 January 2026 (Registration ID: osf.io/a8kdh) and is publicly available (Registration DOI: 10.17605/OSF.IO/A8KDH) [10]. The review was conducted systematically using pre-specified eligibility criteria, data charting procedures, and synthesis methods, consistent with PRISMA-ScR guidance. Reporting follows the 2018 Preferred Reporting Items for Systematic Reviews and Meta-Analyses extension for Scoping Reviews (PRISMA-ScR) [11].

### 2.2. Eligibility Criteria

Studies were selected based on the following criteria in accordance with the Population–Concept–Context framework:

**Inclusion Criteria**

- Population/Context. Studies involving patients in ECMO, ECMO-hybrid or ECMO-adjacent support across adult, pediatric, and neonatal populations.
- Concept. Studies evaluating alternative or novel anticoagulation strategies relative to the standard of care, including but not limited to direct thrombin inhibitors, factor Xa inhibitors, antiplatelets, regional anticoagulation, heparin-free strategies, biocompatibility, and monitoring strategies.
- Outcomes. Studies reporting anticoagulation-related outcomes, including bleeding events, thrombosis, circuit-related events, mortality, and monitoring-related values.
- Types of sources. Randomized controlled trials, observational studies, case series, prospective and retrospective cohort studies were included. The gray literature (clinical trial records, conference abstracts, poster sessions, and reports) was included to capture emerging evidence.
- Timeframe. The search was limited to publications from 2016 onward to capture contemporary developments in ECMO anticoagulation strategies over the most recent decade (2016–2026). This period encompasses major shifts in clinical practice and research, including updated ELSO guidance [12], increased adoption of heparin-sparing or anticoagulation-free protocols [13], expanded use of advanced monitoring techniques [13], and innovations in circuit modification beyond heparin coatings [14]. Furthermore, the COVID-19 pandemic prompted the exploration of alternative anticoagulation strategies in complex ECMO populations [15].

**Exclusion Criteria**

- Narrative or systematic reviews, meta-analyses, editorials, commentaries, opinion pieces, and case reports.
- Studies focused solely on non-ECMO extracorporeal devices (LVAD, Impella, etc.)
- Studies evaluating heparin-only or heparin-optimization strategies—including dosing intensity, antithrombin supplementation, standard monitoring approaches (aPTT, anti-Xa, and ACT), and low-molecular-weight heparin.
- Non-English language publications due to resource limitations.
- Pre-clinical evidence (in vitro, ex vivo, and animal model studies). The review focuses on clinical evidence and registered clinical trials to best characterize the translational and clinical applications of emerging strategies.
- Preprints were excluded from the gray literature.

### 2.3. Information Sources

The literature search was conducted in PubMed (MEDLINE), the Cochrane Library, ClinicalTrials.gov, and the World Health Organization International Clinical Trials Registry Platform (WHO ICTRP) from 1 January 2000 to present. These sources were selected to ensure comprehensive coverage of the peer-reviewed clinical literature and ongoing or recently completed trials evaluating alternative anticoagulation strategies in ECMO.

The gray literature sources included registered clinical trials from ClinicalTrials.gov and WHO ICTRP, as well as manual searches of conference abstracts and poster proceedings from ELSO, SCCM, ASAIO, *Perfusion*, and the *Journal of Thrombosis and Haemostasis* (ISTH). All abstracts and trial records were screened using the same eligibility criteria as peer-reviewed publications. The lack of access to Embase and Web of Science is acknowledged as a limitation. The final search was completed on 8 January 2026.

### 2.4. Search Strategy

A comprehensive search strategy was developed using controlled vocabulary (MeSH terms) and free-text keywords related to ECMO and anticoagulation in PubMed (MEDLINE), Cochrane Library, ClinicalTrials.gov, and WHO ICTRP. The full search strategy is provided in Supplementary Materials S1.

Keywords were combined using Boolean operators (AND, OR), truncation (e.g., anticoagulant), and phrase searching (e.g., “Extracorporeal Membrane Oxygenation”) to capture synonyms, word variants, and exact phrases. The search was structured into three conceptual categories: ECMO, anticoagulation, and alternative strategies. Terms within each category included synonyms and derivative forms (e.g., “Extracorporeal Membrane Oxygenation” OR ECMO OR “extracorporeal life support” OR ECLS). The alternative strategies category included common and emerging anticoagulants (e.g., “Direct Thrombin Inhibitors” OR “factor Xa inhibitor” OR “antiplatelet”). Slight modifications were applied to adapt the search syntax for each database. Restrictions applied included English-only studies and studies published from 2016 onward.

Manual searches of conference proceedings and abstracts were conducted to identify emerging strategies not captured through database searching. Journals and professional organizations were selected based on their established relevance in the ECMO field and research dissemination, through their frequent appearance in the PubMed and Cochrane search results.

### 2.5. Selection of Sources

**Figure 1:**
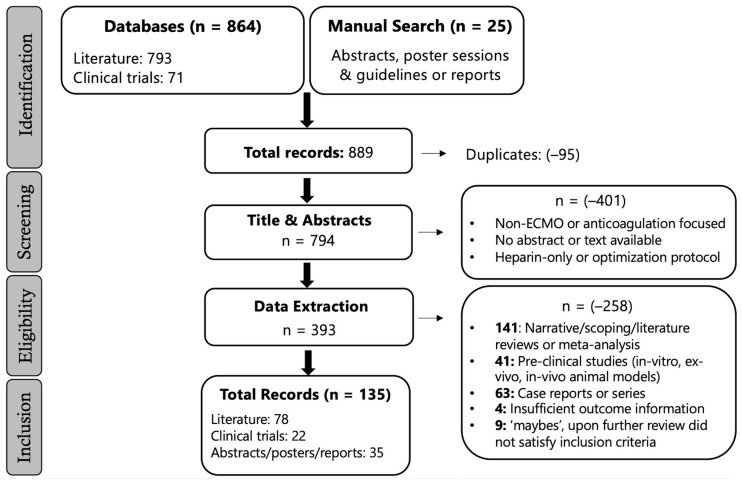
PRISMA study selection process flow diagram.

All records identified were screened by a single reviewer. Titles and abstracts were initially screened for topic relevance, followed by full-text screening where full texts were accessible. When full texts were unavailable, study selection and data extraction were based on the information provided in the abstract. Screening was conducted using predefined eligibility criteria (see Section 2.2). The gray literature sources were screened using the same eligibility criteria. Manual searching of conference abstracts was limited to 2020 onward, as abstracts published prior to 2020 were largely seen as peer-reviewed articles through database searching. Abstracts from 2020 to 2025 were therefore prioritized to capture emerging, unpublished evidence. Records with uncertain eligibility (‘maybe’) were reviewed in consultation with a team member. The study selection process is illustrated in Figure 1.

### 2.6. Data Charting and Synthesis

Data were charted by one reviewer using a standardized data extraction form developed in Google Sheets. The form was piloted on a subset of included studies and refined iteratively to ensure consistency and completeness of the extracted data.

#### 2.6.1. Extracted Variables

Extracted variables included bibliographic information (author and year); study characteristics (design, geographic location, clinical setting, sample size, and single center vs. multicenter); patient characteristics (age group and ECMO indication); ECMO characteristics (VV, VA or hybrid); anticoagulation strategy (pharmacologic, anticoagulation-free, circuit-modification, or monitoring method); and reported outcomes (bleeding, thrombotic events, circuit complications, and mortality). The finalized data charting form, including definitions and examples for each variable, is provided in Supplementary Materials S2.

#### 2.6.2. Synthesis Process

The findings were synthesized descriptively. The peer-reviewed literature and gray literature were analyzed and presented separately. Peer-reviewed clinical studies were organized thematically by strategy type, including pharmacologic agents, anticoagulation-free and biocompatibility approaches, and monitoring or modeling advances. The gray literature was subdivided into registered clinical trials and conference abstracts or poster presentations. Clinical trials were organized by country or geographic location and chronologically by registration date, while abstracts were organized chronologically by author and year of presentation or publication.

## 3. Results

### 3.1. Identification and Screening Process

The results from the database search included 864 combined literature and trials that were identified as eligible for title and abstract screening. After removing 95 duplicates, 889 titles and abstracts remained for screening with a focus on relevance to ECMO anticoagulation. A total of 394 studies were eligible for data extraction consideration. Upon further full-text review and outcome evaluations, studies with insufficient information or those categorized as reviews, case reports, or commentaries were excluded. In total, 78 peer-reviewed articles, 22 clinical trials, and 35 abstracts and proceedings were included for data extraction. Specific exclusions are detailed in the flow chart in Figure 1.

### 3.2. Characteristics of Included Studies

The detailed characteristics of all included studies are presented in Supplementary Materials S3 Tables S1–S3. Each table represents a category of strategy being evaluated: pharmacologic agents (Supplementary Materials S3 Table S1), anticoagulation-free and biocompatibility (Supplementary Materials S3 Table S2), and monitoring and modeling advances (Supplementary Materials S3 Table S3). A total of 153 records were included, comprising 78 peer-reviewed clinical studies and 57 gray literature records (Supplementary Materials S3 Tables S4 and S5).

Study designs included observational, randomized trials, prospective and retrospective cohorts, and case–control studies. Overall, 66 pharmacologic anticoagulation studies were identified. Agents were categorized by mechanism of action, grouped into direct thrombin inhibitors (DTIs), serine protease inhibitors, and others. Within the DTI category, 49 bivalirudin [16,17,18,19,20,21,22,23,24,25,26,27,28,29,30,31,32,33,34,35,36,37,38,39,40,41,42,43,44,45,46,47,48,49,50,51,52,53,54,55,56,57,58,59,60,61,62,63,64] and nine argatroban [65,66,67,68,69,70,71,72,73] studies were included. Among the serine protease inhibitors, five nafamostat mesilate studies [74,75,76] were recorded. The ‘other’ category included factor pathway inhibitors [77,78], prostaglandin E1 [79], P2Y12 inhibitor [80], and regional citrate anticoagulation [81]. Five anticoagulation-free or heparin-sparing studies [82,83,84,85,86] and one biocompatible study were identified [87]. Lastly, six monitoring and modeling advancement studies were included [88,89,90,91,92,93].

From the gray literature, registered clinical trials ranged from 2016 to 2024, with China and Austria contributing the greatest number of trials, followed by Italy and Canada. Among conference abstracts, proceedings and reports, the year 2022 had the highest number of studies focused on anticoagulation strategies.

Overall, VV-ECMO was more frequently studied than VA-ECMO, with few studies including hybrid modalities (e.g., ECpella, VV-PECLA, concurrent CVVH, etc.). The adult population predominated the literature; neonatal and infant populations were underrepresented. Single-center retrospective cohort study designs, typically using electronic medical records or center-specific registry data, were the most common study designs.

### 3.3. Individual Evidence

The evidence is presented by anticoagulation strategy, with summary tables provided at the end of each strategy section (Table 1, Table 2 and Table 3).

#### 3.3.1. Pharmacologic Agents

##### Direct Thrombin Inhibitors

**Bivalirudin**

The majority of bivalirudin studies were single-center retrospective cohort analyses, with comparative and observational designs predominating over case–control and matched-control approaches. Adults were the most frequently studied population, followed by pediatric patients. In the past year, three neonatal-focused studies emerged, including one prospective pilot study in neonates undergoing arterial switch repair [16,18,22]. Several studies included mixed populations: four combined pediatric and neonatal patients, one included adults and pediatric patients, and one encompassed neonates, pediatric patients, and adults. Five multicenter studies were identified [34,36,42,47,48]: three in adults and two in pediatric patients (<18 years). Two multicenter studies evaluated COVID-19 patients treated with DTIs (bivalirudin or argatroban), with one focusing on VV-ECMO patients with COVID-19-associated ARDS (CARDS) [47,48].

Approximately half of the studies did not specify ECMO modality. Among those that did, thirteen evaluated both VV and VA-ECMO, eight focused exclusively on VV-ECMO, three on VA-ECMO [33,40,60], and two on hybrid configurations [16,17]. All modality-specific studies were conducted in adults (≥18 years), except one VA-ECMO study [60]. VV-ECMO studies primarily involved ARDS, COVID-19 infection, and CARDS, whereas VA-ECMO studies focused on cardiogenic shock and cardiac arrest. Of the studies reporting geographic location, nine were conducted in the United States, two in Turkey [25,32], one in India [16], and one in Italy [45]. The clinical settings included eleven tertiary care centers, two quaternary pediatric hospitals [22,24], one primary ECMO center [21], and four perioperative settings [16,18,24,60].

UFH was the most common comparator. Two studies evaluated bivalirudin with aspirin [23,32], one with bivalirudin, cangrelor and aspirin [31], and one with bivalirudin and cangrelor alone [33]. Nine studies focused on bivalirudin monitoring, mostly in pediatric and neonatal populations. Refs. [27,28,30,34,41,46,49,60,61], and two pediatric studies evaluated bivalirudin administration with continuous renal replacement therapy [42,44].

Most studies assessed safety and feasibility outcomes, including dosing adjustments, percent time in therapeutic range, and monitoring assay performance (e.g., TEG, ACT, aPTT, PTT, and INR). Commonly thrombotic complications included deep vein thrombosis (DVT), in-circuit thrombosis, pulmonary embolism (PE), and ischemic stroke. Bleeding outcomes included major and minor bleeding, intracranial hemorrhage (ICH), and gastrointestinal (GI) bleeding. Few studies used standardized definitions for bleeding events (ELSO, BARC, or GUSTO). Additional outcomes included ICU and in-hospital mortality, circuit-related events, and transfusion requirements. A small number of studies reported cost-analyses [25,38,50,51,56] and neurological outcomes [19,36,64].

**Argatroban**

Argatroban studies represented approximately one-fifth of the number of bivalirudin studies. Among the nine identified, five were single-center retrospective cohort studies, including one observational matched-control design [73]. Three were observational studies, including one prospective design and one non-inferiority, propensity score-matched analysis [66,67,72]. One study was multicenter, using a bi-centric exploratory cohort design [67]. Six studies focused exclusively on VV-ECMO, one evaluated both VV and VA-ECMO [70], one examined a hybrid VV-ECMO pumpless extracorporeal lung assist (PECLA) device [73], and one did not specify the ECMO modality [72]. All studies were conducted in adult populations. Among VV-ECMO-only and VV-PECLA studies, the primary indications were severe ARDS and CARDS [65,66,67,68,69,71,73].

Of the eight studies reporting geographic location, seven were conducted outside the United States: three in Germany, two in the Czech Republic, one in Austria, and one in Italy. Six studies were conducted within university hospital ICUs, one in a tertiary care center [67], and one in a community hospital [70]. Only one study evaluated ECMO anticoagulation in a perioperative setting [72]. UFH was the sole comparator across all studies. Two studies specifically assessed argatroban monitoring strategies [65,67], and one evaluated argatroban administration with CRRT [72].

Overall, reported outcomes most frequently addressed safety and feasibility, including dosing adjustments, percent time in therapeutic range, and the performance of monitoring assays (e.g., aPTT, PT, dTT, antti-Xa, and anti-IIa). Common bleeding complications included ICH, major and minor bleedings, GI bleeding, and pulmonary bleeding, though few studies applied standardized definitions using ELSO and BARC criteria. Frequently reported thrombotic events included circuit thrombosis, DVT, PE, and ischemic events. Other commonly evaluated outcomes included transfusion requirements and mortality. A small number of studies also evaluated total costs associated with argatroban versus UFH administration [70,71].

##### Serine Protease Inhibitors

All studies evaluating nafamostat mesylate (NM) were retrospective, with the most recent published in 2022 [74]. All three were in single-center studies in adult populations. Two focused exclusively on VA-ECMO, while one included both VV and VA-ECMO. Indications for VA-ECMO included post-cardiotomy support and cardiovascular or respiratory disease. All studies were conducted in South Korea in perioperative settings. UFH was the sole comparator across studies [74,75,76], and only one evaluated NM administration with CRRT [74].

All studies assessed bleeding and thrombotic complications. Common bleeding events included cerebral hemorrhage, bleeding requiring intervention, and GI bleeding. Frequently reported thrombotic complications included intracardiac thrombus and embolic stroke. Two studies reported median ECMO duration, and one additionally examined safety, feasibility and transfusion requirements [75].

##### Other

Within this category, two factor pathway inhibitor studies were identified, one retrospective observational study [78] and one prospective cohort study [77], both conducted in adult ICU populations—one in Austria and the other in Japan. The prospective cohort evaluated factor XII (FXII) activity and its association with thromboembolic and bleeding complications [77]. The retrospective observational study evaluated the safety and feasibility of recombinant human thrombomodulin (rhTM), either alone or in combination with UFH or antithrombin (AT) [78]. Both studies reported bleeding and thrombotic outcomes.

One multicenter, randomized, double-blind, phase 2 pilot trial conducted in Austria evaluated prostaglandin E1 (PGE1), alone or combined with UFH, in adult VV-ECMO patients [79]. The primary outcome was bleeding rate, while secondary outcomes included incidence and time to clinically overt bleeding (BARC), thrombotic events (PE and DVT), and mortality. The remaining two retrospective studies assessed alternative strategies: cangrelor in cariogenic shock patients supported on VA-ECMO or a VA-Impella hybrid configuration, and regional citrate anticoagulation in adult VV-ECMO patients receiving CRRT, either alone or with UFH [80,81]. Both evaluated thrombotic complications.

#### 3.3.2. Anticoagulation-Free and Circuit-Modified Strategies

Of the five studies evaluating anticoagulation-free or heparin-sparing strategies, all were single-center retrospective cohort analyses, including two from China [83,84] and one from the U.S. [82]. All studies focused exclusively on adult populations. There was an equal distribution between VA- and VV-ECMO-only circuits. Two studies used ECMO as a bridge to lung transplantation [82,83], and two were conducted in trauma patients [84,86]. Strategies included heparin-free approaches [83,84], heparin-sparing strategies using heparin-coated circuits with low-dose or no systemic anticoagulation [82,86], and one using no systemic anticoagulation without circuit modification [85]. Common outcomes included circuit-related events, transfusion requirements, thrombotic and bleeding complications, and mortality.

Among clinical applications of biocompatible or circuit-modified systems, only one study was identified: a multicenter, retrospective, propensity score-weighted cohort analysis across three ECMO centers evaluating adults with severe cardiogenic shock on VA-ECMO [87]. The study compared phosphorylcholine-coated circuits with heparin-coated circuits in their ability to reduce bleeding complications, thrombus formation, and mortality [87].

#### 3.3.3. Monitoring and Modeling Advances

Of the six identified studies, three were single-center retrospective cohort analyses: one was a multicenter prospective randomized controlled pilot trial, one was a single-center prospective observational study, and one was a quantitative modeling exploratory study. Two focused exclusively on pediatric populations [89,90], and one included both pediatric and neonatal patients [93]. Two studies were conducted outside of the U.S. (Canada and Italy) [88,92], and two were conducted in a tertiary care center [92,93]. Only one study exclusively evaluated VV-ECMO in patients with ARDS or as a bridge to lung transplant [92].

Two studies assessed correlations between thromboelastography (TEG) parameters and standard coagulation assays for UFH monitoring [92,93], and one compared rotational thromboelastometry (ROTEM) with TEG and other conventional assays [91]. Another study evaluated the anticoagulation effects of bivalirudin and UFH using a Factor IIa Clotting Time Score [90]. The more recent studies incorporated quantitative and modeling-based approaches: one predicted optimal therapeutic dosing, while another modeled associations between aPTT trajectories and adverse clinical events using baseline patient data [88,89]. Together, these studies highlight the growing interest in advancing anticoagulation monitoring through quantitative, modeling, and data-driven approaches to optimize anticoagulation management in ECMO.

#### 3.3.4. Gray Literature

Among the 22 registered clinical trials, ten are actively recruiting, eight have been completed, and one was terminated. Two trials are in Phase 1, four in Phase 2, and three in Phase 4. Most are interventional randomized controlled trials, followed by prospective observational designs. Sponsors were primarily university hospitals, followed by health institutes or non-university health systems. Most trials focus on adult populations: one included neonates and infants (CTRI/2024/11/077189), and one targeted children under 10 years old (JPRN-jRCT1012220032). Only three trials were multicenter.

Of trials reporting ECMO modality, six focused exclusively on VV-ECMO, five evaluated both VV and VA-ECMO, one focused on VA-ECMO alone (NCT06792643), and one examined a hybrid configuration (CTRI/2024/11/077189). Investigated pharmacologic agents included bivalirudin (four trials: one in the U.S, two in Asia, and one in Australia), argatroban, (six trials, primarily in Europe and Asia), prostaglandin EI (PGE1; two trials in Austria), nafamostat mesylate (three trials in China), and cangrelor (one trial in Italy). Four trials evaluated monitoring strategies: two using TEG (JPRN-jRCT1012220032, NCT04268940) and two employing novel methods (NCT03832842, NCT03815773). Commonly evaluated outcomes include bleeding events (defined by BARC, WHO, ELSO or ISTH criteria), thrombotic complications, mortality, transfusion requirements, and safety and feasibility.

Among abstracts and conference proceedings, most were single-center retrospective studies, followed by prospective and observational designs. Only one open-label RCT and four multicenter studies were identified. Most focused on adults, while five focused on neonates, five on pediatric patients, and four included mixed populations. Only one study evaluated peripartum patients. Most evaluated both VA and VV-ECMO, followed by VV-ECMO alone. Direct thrombin inhibitors—particularly bivalirudin—were the most frequently studied anticoagulation strategies, followed by anticoagulation-free approaches and monitoring-focused strategies. Most studies were conducted in the U.S., with others from Europe and Asia. Common outcomes included bleeding events (BARC criteria), thrombotic events, circuit-related complications, and safety and feasibility measures.

### 3.4. Synthesis of Results

Evidence on novel or alternative anticoagulation strategies in ECMO is heterogeneous, varying by study design, sample size, patient population, ECMO modality, and anticoagulation approach. A total of 135 records were included, comprising peer-reviewed and gray literature. Adults were the most frequently studied population, while neonatal patients were underrepresented. VV-ECMO-focused studies were more common than VA-ECMO, and retrospective cohort designs predominated.

Among pharmacologic strategies, bivalirudin was the most frequently studied across diverse designs and patient populations, primarily in the U.S. Anticoagulation-free and heparin-sparing strategies often combined low-dose systemic anticoagulation with heparin-coated circuits, typically applied as a bridge to transplant patients. Clinical evidence for biocompatible and circuit-modified systems remains limited. Monitoring strategies such as TEG and ROTEM continue to be evaluated, with emerging approaches including Factor IIa Clotting Time Scores and predictive quantitative models linking aPTT trajectories to anticoagulation dosing and outcomes.

**Figure 2:**
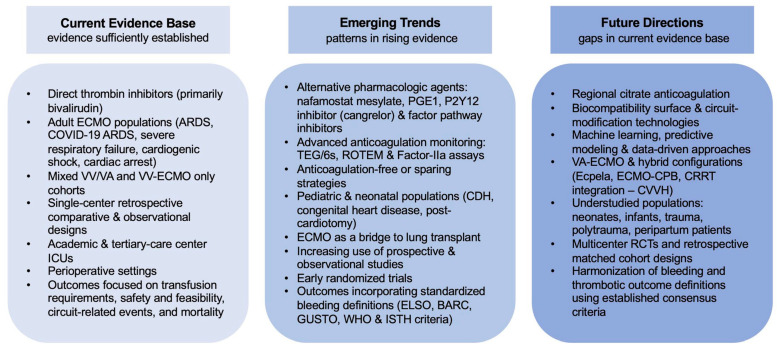
Conceptual summary of the current alternative anticoagulation strategies in the ECMO evidence base, highlighting areas of sufficiently established evidence, emerging trends driven by rising evidence and patterns, and future directions informed by gaps in the existing literature.

The gray literature, including registered clinical trials, is largely interventional and focuses on bivalirudin and argatroban, followed by PGE1 and NM, in adult populations. Recent trials increasingly include neonatal and pediatric populations. Conference abstracts reflect similar trends, predominantly evaluating bivalirudin and argatroban in adults on VV and VA-ECMO, with anticoagulation-free or monitoring strategies less represented. Overall, bivalirudin remains the most extensively evaluated pharmacologic alternative to UFH, alongside emerging anticoagulation-free, biocompatible or circuit-modifying strategies. Gaps persist in pediatric and neonatal populations, multicenter randomized control trials or prospective studies, and innovative monitoring tools. Figure 2 summarizes established and emerging evidence and highlights future directions.

## 4. Discussion

This scoping review mapped the current evidence on novel and alternative anticoagulation strategies in ECMO, a field that is transitioning from heparin-centered toward more diversified and personalized anticoagulation approaches tailored to patient and center needs. Among the pharmacologic alternatives identified, DTIs—particularly bivalirudin—emerged as the most clinically integrated and evidence-supported alternatives to UFH, followed by NM. This finding aligns with the existing literature [5,6,7,8]. The presence of DTI’s across clinical studies, the gray literature, and official guidelines reflects their growing acceptability as not only alternatives but potential primary anticoagulants in real-world ECMO practice [12,94,95], suggesting a transition from second-line to first-line use in selected populations.

Beyond DTIs, emerging pharmacologic agents such as PGE1, P2Y12 inhibitors, and factor pathway inhibitors (e.g., FXa and FII) remain largely exploratory, with evidence limited to a small number of clinical studies. This observation is consistent with prior reviews examining alternative ECMO anticoagulation strategies [3,4,9,96]. The geographic concentration of ongoing clinical trials—NM and bivalirudin in Asia, and PGE1 and argatroban in Europe—highlights regional variability in anticoagulation innovation and adoption, underscoring the pharmacologic diversification occurring within the ECMO field.

The predominance of retrospective, adult-focused, single-center studies highlights essential limitations in the existing evidence base informing ECMO anticoagulation practice. While these studies provide valuable foundational data, their limited generalizability and lack of standardization remain significant challenges. Thus, many urge for an increase in multicentered RCTs to reduce heterogeneity in anticoagulation protocols, dosing strategies, and monitoring approaches across diverse patient populations [3,9,12,97]. In the absence of higher-quality evidence, the development of robust, evidence-based guidelines remains hampered, and institutional practice variations are likely to persist—particularly in underrepresented populations [97]. Circuit- and surface-based innovations represent a promising yet under-translated area of ECMO anticoagulation research, remaining largely confined to pre-clinical or exploratory phases and requiring further clinical evidence and validation before widespread implementation [98].

Advances in anticoagulation monitoring represent another evolving component of ECMO management. Traditional laboratory assays (e.g., aPTT and ACT) and viscoelastic testing (e.g., ROTEM and TEG) remain integral to anticoagulation—especially in DTIs—despite the lack of consensus regarding optimal monitoring strategies [7,8]. The emerging approaches extend beyond indirect systemic measurements and toward direct assessment of circuit-level pathology, predictive analytics, and real-time evaluations of coagulation dynamics [96,97]. Quantitative modeling and machine learning techniques suggest a future in which anticoagulation management will be guided by patient-specific predictive frameworks [99].

Despite these advances, outcome assessment across studies remains limited by heterogeneity in bleeding and thrombotic event definitions, as well as variability in institutional protocols. Although the ELSO provides standardized definitions, inconsistent application, or substitution with alternative criteria such as BARC, WHO, or ISTH, continues to restrict cross-study comparability and generalizability. This variability highlights the need for greater consensus on core outcome definitions specific to ECMO anticoagulation research.

Overall, these findings indicate that ECMO anticoagulation is evolving with three main focuses: pharmacologic optimization, led by DTIs as the most clinically investigated alternatives to UFH; device-level innovation, driven by pre-clinical advances in circuit biocompatibility; and data-driven monitoring, emphasizing prediction, personalization, and precision-based anticoagulation management.

### Limitations

This review has several limitations. Database coverage and access may have restricted the breadth of studies identified. The lack of access to Embase, Scopus, and Web of Science may have resulted in missed studies, particularly those originating from Europe and Asia, where pharmacologic and device-based ECMO anticoagulation research is actively conducted. Additionally, the inclusion of English-only publications introduces potential language and publication bias, and may underrepresent evidence published in non-English journals. As this was a scoping review, no formal quality appraisal was conducted; therefore, the findings are intended to map the existing evidence and identify trends rather than assess the effectiveness, risk of bias, or strength of evidence. Screening, study selection, data extraction, and categorization were performed by a single reviewer, which may introduce misclassification and data extraction errors. Lastly, because the protocol was not established a priori, there is a potential risk of selection and reporting bias.

## 5. Conclusions

This scoping review maps the evolving landscape of ECMO anticoagulation beyond UFH, showing that direct thrombin inhibitors are the most established alternatives. In contrast, agents such as nafamostat mesylate, prostaglandin E1, and factor pathway inhibitors remain in early clinical investigation and require further validation before wider adoption. Across the peer-reviewed and gray literature, the evidence is highly heterogeneous. Most clinical data remain retrospective and adult-centered, with limited multicenter randomized trials. Emerging monitoring approaches, biocompatible circuit technologies, and machine learning models reflect a shift toward more individualized and mechanistic management, although these innovations are still largely in early development. Overall, the field is transitioning from reliance on a single anticoagulant toward more diversified and personalized strategies. Future research should prioritize multicenter randomized trials, standardization of protocols, and integrate pharmacologic, circuit-based, and monitoring innovations to meet patient needs.

## Figures and Tables

**Table 1:** Summary of data extracted by pharmacologic agent, highlighting the most established evidence for each variable of interest across included studies (*n* = 66). A dash (-) means not indicated or specified in the study’s abstract or full text.

Pharmacologic Agent (*n*)	Study Design	Setting	Location	Sample Size Range	Population	ECMO Type	Outcomes
Bivalirudin [49]	Single-center retrospective cohorts	Tertiary care center ICU	USA	10–570	Adults	VV & VA	Safety & feasibility, bleeding & thrombotic events
Argatroban [9]	Single-center retrospective cohorts	University hospital ICU	Europe	40–117	Adults	VV	Safety & feasibility, bleeding & thrombotic events
Nafamostat Mesylate [3]	Single-center retrospective cohorts	Perioperative period in ICU	South Korea	16–320	Adults	VA	Bleeding & thrombotic events
Prostaglandin E1 [1]	Multicenter randomized controlled trial	University hospital ICU	Austria	48	Adults	VV	Bleeding & thrombotic events, transfusion requirements
Factor Pathway Inhibitors [2]	Single-center prospective/retrospective observational	University hospital ICU	Austria & Japan	15–51	Adults	VV/	Safety & feasibility
						VV & VA	
Cangrelor [1]	Single-center retrospective observational	Hospital ICU	-	17	-	VA	Bleeding & thrombotic events
Regional Citrate Anticoagulation [1]	Retrospective	Hospital ICU	-	48	Adults	VV	Safety & feasibility, bleeding & thrombotic events

**Table 2:** Summary of data extracted for anticoagulation monitoring strategies, highlighting the most established evidence for each variable across included studies (*n* = 6). A dash (-) means not indicated or specified in the study’s abstract or full text.

Monitoring & Modelling Strategies	Study Design	Setting	Location	Sample Size Range	Population	ECMO Type	Outcomes
Monitoring strategies [4]	Single-center retrospective/prospective	Tertiary care center	USA & Italy	15–42	Adults/pediatric	VV & VA	Safety & feasibility
Modeling approaches [2]	Quantitative methods & survival modeling	-	Canada	109	Adults/pediatric	-	Bleeding complications, therapeutic dose

**Table 3:** Summary of the gray literature, highlighting the most established evidence for each variable across registered clinical trials, abstracts, poster presentations, and reports (*n* = 57).

Gray Literature (*n*)	Study Design	Strategy	Location	Sample Size Range	Population	ECMO Type	Outcomes
Clinical Trials [22]	Single-center interventional RCT	DTIs	Austria & China	4–656	Adults	VV	Safety & feasibility, bleeding & thrombosis events, transfusion requirements, mortality
Abstracts, posters & reports [35]	Single-center retrospective	DTIs	U.S.	3–1542	Adults	VV & VA	Safety & feasibility, bleeding & thrombosis events, circuit-related events

## Data Availability

No new data were created or analyzed in this study.

## Supplementary Materials

The following supporting information can be downloaded at: https://www.mdpi.com/article/10.3390/jcm15062337/s1, Supplementary Material S1: Full search strategy for literature review; Supplementary Material S2: The finalized data charting form with definitions and examples for each extracted variable; Supplementary Material S3: Data extraction of included studies, presented in Tables S1–S5, each corresponding to a specific anticoagulation strategy category; Table S1: Pharmacologic agent strategies in ECMO anticoagulation. Studies are categorized by agent and presented in chronological order by author and year. The number of included studies per agent is as follows: bivalirudin (*n* = 49), argatroban (*n* = 9), nafamostat mesylate (*n* = 3), and other agents (*n* = 5); Table S2: Anticoagulation-free or anticoagulation-sparing and biocompatibility strategies, presented by category and arranged in chronological order by author and year. The number of included studies per category is as follows: anticoagulation-free or anticoagulation-sparing (*n* = 5), and biocompatibility or circuit-modifying strategies (*n* = 1); Table S3: Monitoring and modeling strategies, arranged in chronological order by author and year. The number of included studies is (*n* = 6); Table S4: Identified clinical trials organized by country in alphabetical order; countries with multiple trials are further arranged by trial start year (*n* = 22); Table S5: Identified abstracts, poster sessions, and guidelines from 2020 to 2025, organized chronologically by author and year (*n* = 35).

## Abbreviations

ARDS	Acute respiratory distress syndrome
DTIs	Direct thrombin inhibitors
ECMO	Extracorporeal membrane oxygenation
ICU	Intensive care unit
ROTEM	Rotational thromboelastometry
TEG	Thromboelastography
UFH	Unfractionated heparin
VA	Venoarterial
VV	Venovenous
VAD	Ventricular assist device
vWF	von Willebrand factor
