# Supplementary material for: Alternate and Emerging Anticoagulation Strategies for Extracorporeal Membrane Oxygenation: A Scoping Review
Source: J Clin Med. 2026 Mar 18;15(6):2337. doi: 10.3390/jcm15062337 (PMC13027120; doi:10.3390/jcm15062337)
Supplement: Supplementary file 1 [file jcm-15-02337-s001.zip › jcm-4046763-supplementary.pdf]

Supplementary Material S1. Full search strategy for literature review

Keywords	Search Date	Search Engine	Result	Total Included
("Extracorporeal Membrane Oxygenation"[MeSH]<br>OR ECMO[tiab]<br>OR "extracorporeal life support"[tiab]<br>OR ECLS[tiab])<br>**AND**<br>("Anticoagulants"[MeSH]<br>OR "Anticoagulation"[MeSH]<br>OR anticoagulation[tiab]<br>OR anticoagulant*[tiab]<br>OR antithrombotic*[tiab]<br>OR hemostasis[tiab]<br>OR "coagulation management"[tiab])<br>**AND**<br>(("Direct Thrombin Inhibitors"[MeSH]<br>OR bivalirudin[tiab]<br>OR argatroban[tiab]<br>OR desirudin[tiab]<br>OR lepirudin[tiab])<br>OR<br>("factor Xa inhibitor*"[tiab]<br>OR fondaparinux[tiab]<br>OR DOAC*[tiab]<br>OR apixaban[tiab]<br>OR rivaroxaban[tiab])<br>OR<br>(antiplatelet*[tiab]<br>OR aspirin[tiab]<br>OR "P2Y12 inhibitor*"[tiab]<br>OR "platelet inhibition"[tiab])<br>OR<br>("serine protease inhibitor*"[tiab]<br>OR "factor XII inhibitor*"[tiab]<br>OR nafamostat[tiab]<br>OR gabexate[tiab]<br>OR "contact pathway inhibition"[tiab])<br>OR<br>("heparin-bonded circuits"[tiab]<br>OR "biocompatible circuits"[tiab]<br>OR "surface-modified circuits"[tiab])<br>OR<br>("low-intensity anticoagulation"[tiab]	1/2/2026	Pubmed	636	78

OR "reduced anticoagulation"[tiab]<br>OR "anticoagulation-free ECMO"[tiab])<br>OR<br>(heparin[tiab]<br>OR "unfractionated heparin"[tiab]<br>OR "non-heparin"[tiab]<br>OR "heparin-free"[tiab]<br>OR alternative*[tiab]<br>OR novel*[tiab]))				
((Extracorporeal NEXT membrane NEXT oxygenation*) OR ECMO OR (extracorporeal NEXT life NEXT support*) OR ECLS)<br>**AND**<br>(anticoagulation OR anticoagulant* OR antithrombotic* OR hemostasis OR (coagulation NEXT management*))<br>**AND**<br>(heparin OR (non-heparin*) OR (heparin-free*) OR alternative* OR novel* OR bivalirudin OR argatroban OR desirudin OR lepirudin OR (factor NEXT Xa NEXT inhibitor*) OR fondaparinux OR DOAC* OR apixaban OR rivaroxaban OR nafamostat OR gabexate OR antiplatelet* OR aspirin OR "P2Y12 NEXT inhibitor*"OR "platelet NEXT inhibition"OR "serine NEXT protease NEXT inhibitor*" OR "factor NEXT XII NEXT inhibitor*" OR "contact NEXT pathway NEXT inhibition" OR "surface-modified NEXT circuits" OR "biocompatible NEXT circuits" OR "heparin-bonded NEXT circuits" OR "low-intensity NEXT anticoagulation" OR "reduced NEXT anticoagulation" OR "anticoagulation-free NEXT ECMO")	1/3/2026	Cochrane Library	157	26
(ECMO OR "extracorporeal membrane oxygenation") **AND**<br>(anticoagulation OR anticoagulant)	1/2/2026	ClinicalTrials.gov	31	7
(ECMO **AND** anticoagulation), ("extracorporeal membrane oxygenation" **AND** anticoagulation)	1/8/2026	WHO ICTRP	40	2

Limits: English-only, from 2016 to 2026 (present)
Last Date Searched: Jan 8, 2026

Supplementary Material S2. The finalized data charting form with definitions and examples for each extracted variable.

Outcome definitions followed those reported by the original studies. When definitions were not provided, outcomes were extracted based on the descriptions in the abstract (if full text was unavailable) or in the main text. The following charting form was adapted for grey literature (clinical trials and abstracts/poster sessions) to accurately reflect the information available.

**Variable**	**Definition**	**Example**
Author, year	First author and publication year	*Badge et al,* 2025
Study design	Type of study conducted	Prospective pilot cohort study
Single vs Multicenter	Number of institutions or centers involved in the study – single-center (n=1) vs multicenter (n= +2)	Single center
Clinical setting / Location	Type of center or hospital, department, unit, or period the ECMO treatment is being administered.<br><br>Physical geographic place the study was conducted	Quaternary pediatric hospital, pediatric and neonatal intensive care units (ICUs)<br><br>Dallas, TX, USA
Patient Population	Age group of the included patient population (e.g., neonatal, pediatric, adult)	Neonatal
Sample Size (n)	Total of included patients in study	30
ECMO modality	Type of ECMO circuit used: VV, VA or hybrid	VA
Indication for ECMO	Disease(s) or diagnoses prompting ECMO use.	Cardiogenic shock
Anticoagulant strategy (intervention)	Primary anticoagulant agent or strategy being implemented and investigated.	Pharmacologic agent – DTI, Bivalirudin<br><br>Bolus dose of 1 mg/kg, with a maintenance dose of 2.5 mg/kg/h
Anticoagulant strategy: comparator (if applicable)	Reference to the intervention, typically the standard of care	Unfractionated heparin (UFH)
Outcome Measures: bleeding complications	Any reported hemorrhagic event occurring during ECMO support:<br>• major bleeding<br>• minor bleeding<br>• intracranial hemorrhage<br>• gastrointestinal bleeding<br>• surgical site bleeding<br>• bleeding requiring clinical intervention (e.g., transfusions, circuit change, or dose adjustments.)	Intracranial hemorrhage
Outcome Measures: thrombotic events	Any reported thrombotic event affecting the patient or the ECMO system:<br>• circuit thrombosis<br>• oxygenator clotting<br>• pump thrombosis<br>• deep vein thrombosis	Circuit thrombosis

	• pulmonary embolism<br>• ischemic stroke	
Outcome Measures: circuit-related events	Any complications involving the ECMO circuit duration:<br>• oxygenator failure<br>• pump malfunction<br>• circuit or oxygenator exchange<br>• flow impairment<br>• ECMO duration	Oxygenator exchange required (yes/no); # of exchanges
Mortality (if reported)	Any reported death:<br>• in-hospital mortality<br>• intensive care unit mortality<br>• mortality within a specified follow-up period (defined by the study)	ICU mortality
Other measures: transfusion requirements	Any reported use of blood products during ECMO:<br>• packed red blood cells<br>• platelets<br>• fresh frozen plasma<br>• cryoprecipitate<br>Reported as total units, frequency, or proportion of patients requiring transfusion.	# of fresh frozen plasma units
Other measures: safety & feasibility	Reported measures evaluating the safety, practicality, and clinical use of an anticoagulation strategy, including:<br>• anticoagulant-related adverse events or complications<br>• ease & feasibility of monitoring, including:<br>o % time in therapeutic range (%TTR)<br>o Performance of global coagulation assays & viscoelastic testing (e.g. aPTT, ACT, dTT, TEG, ROTEM)<br>o Achievement of goal target ranges<br>• Correlation or predictive performance of monitoring assays in assessing coagulation & anticipating bleeding and thrombotic events<br>• Dosing ranges and adjustments	%TTR

Supplementary Material S3. Data extraction of included studies, presented in Tables S1–S5, each corresponding to a specific anticoagulation strategy category.

Table S1. Pharmacologic agent strategies in ECMO anticoagulation. Studies are categorized by agent and presented in chronological order by author and year. The number of included studies per agent is as follows: bivalirudin (n = 49), argatroban (n = 9), nafamostat mesylate (n = 3), and other agents (n = 5)

Author, year	Study Design	Single vs multicenter	Setting	Population	Size (n)	ECMO Modality	Indication for ECMO	Intervention or Comparator	Outcome measures
**DTI: Bivalirudin**									
Badge et al, 2025	Prospective pilot study<br><br>CTRI:2024/11/077189	Single center	Tertiary care referral hospital<br><br>(India)	Neonates & infants	10	ECMO-CPB circuit	Arterial switch operation, congenital malformation of great arteries	Bolus dose of 1 mg/kg & maintenance dose of 2.5 mg/kg/h<br><br>ACT > 480s	• ACT values<br>• Dose adjustments<br>• Thromboembolic or circuit complications<br>• Transfusions requirements<br>• ICU length of stay (LOS)<br>• Mortality
Cho et al, 2025	Retrospective observational comparative analysis from July 2021 to April 2024)	Single center	Tertiary academic medical center	(18 y.o.+)	98	VA + Impella (ECpella)	Cardiogenic shock (CS)	Bivalirudin vs. Heparin vs. Bivalirudin + heparin	• Frequency of thrombus formation (DVT/aortic)<br>• Mortality<br>• ICU (LOS)
Credille et al, 2025	Retrospective cohort comparative pilot study from 2008 to 2023	Single center	Post-operative period<br><br>(Children's Hospital, Colorado)	Neonates	62	Not specified	Congenital diaphragmatic hernia (CDH) repair	Bivalirudin vs. heparin	• Bleeding events: requiring surgical repair or intracranial hemorrhage (ICH)<br>• Volume of blood product transfusions<br>• # of circuit changes<br>• ECMO duration<br>• Survival
Daniels et al, 2025	Retrospective cohort comparative analysis	Single center	Pediatric cardiothoraci c ICU	Pediatric	122	Not specified	-	Bivalirudin vs heparin	• Thrombotic & bleeding events (major)<br>• Neurologic complications<br>• Survival to decannulation,

									transplant, & discharge<br>• Circuit changes<br>• Blood product transfusion volume
Giorni et al, 2025	Retrospective cohort comparative analysis from February 2018 to August 2020	Single center	Tertiary pediatric cardiac ICU	Pediatric	36	Not specified	Congestive heart failure	Bivalirudin vs. heparin	• Thrombotic episodes<br>• Cerebrovascular events<br>• Mortality<br>• PTT range
Lofy et al, 2025	Retrospective cohort study	Single center	Primary ECMO & tertiary academic medical center	-	-	Not specified	-	Bivalirudin vs heparin	• Bleeding events<br>• Thrombosis rates: systemic & device<br>• Time to therapeutic range (TTR)
McMichael et al, 2025	Prospective Randomized open-label pilot trial<br><br>RCT: NCT03318393 from 2018 to 2021	Single center	Quaternary pediatric hospital<br><br>(Children's Medical Center, Dallas, TX, USA)	Neonatal & pediatric<br><br>(0 days to <18)	30	VV & VA	ARDS, cardiogenic shock, cardiogenic dysfunction, ECPR, post-cardiac bypass, sepsis	Bivalirudin vs heparin	• Time spent in goal anticoagulation<br>• Hemorrhagic complications: ICH, pleural/pericardial, GI, surgical site bleed<br>• Thrombotic events: stroke & circuit<br>• ECMO duration (hr)<br>• Survival to discharge & decannulation<br>• Blood products transfused
Ogus et al, 2025	Retrospective cohort analysis	-	ICU	Adult	70	Not specified	COVID-19 associated ARDS (CARDS)	Bivalirudin + aspirin	• aPTT monitoring: international normalized ratio (INR) & platelet count<br>• In-circuit thrombosis<br>• ECMO duration (days)<br>• Oxygenator changes

Smith et al, 2025	Retrospective cohort chart review from January 2021 to June 2023	Single center	Quaternary center PICU<br><br>(West Virginia University Medicine Children's Hospital, Children's Heart Center)	(< 19 y.o.)<br><br>Neonates-adolescents	14	VV & VA	ECPR, cardiac failure non-postoperative, cardiac failure postoperative, respiratory failure	Bivalirudin	• TTR & time to goal aPTT<br>• ECMO duration (days)<br>• Dose adjustment due to continuous renal replacement therapy (CRRT)<br>• # of blood products transfused<br>• Major & minor bleeding events<br>• Time to circuit change (hrs.)
Yilmaz et al, 2025	Retrospective cohort, -before & after study from March 2013 to 2022	Single center	ICU<br><br>(University of Health Sciences Türkiye, İstanbul Training and Research Hospital, Istanbul, Turkey)	(18 y.o +)	56	VV	ARDS	Bivalirudin vs heparin	• Difference in blood transfusions<br>• Cost-effectiveness<br>• 15-day thrombosis: vascular or circuit<br>• Bleeding events: pericanular, pulmoner, hematuria, hematemesis, other<br>• Time to aPTT target (hrs.)<br>• Total ECMO days<br>• In-hospital mortality
Chomat et al, 2024	Retrospective cohort study from January 2019 to December 2021	Single center	ICU	Children	60<br><br>TPE sessio ns	Not specified	-	Bivalirudin + replacement fluid during therapeutic plasma exchange (TPE)	• Bleeding events<br>• Circuit thrombosis<br>• Dose adjustments & coagulation parameters post TPE<br>• TEG reaction time, clot time, INR, PTT
Engel et al, 2024	Retrospective cohort data from 2018 to 2022	Single center	ICU at Cincinnati Children's Hospital Medical	Children	22	Not specified	-	Bivalirudin - Monitoring	• Correlation between anti-IIa, Bival calibrated dTT, dTT & aPTT monitoring assays

			Center, Ohio, USA)						
Jatis et al, 2024	Retrospective cohort from 2018 to 2022	-	ICU	Adults	214	Not specified	-	Bivalirudin – monitoring	• Assess relationship between dosing & aPTT<br>• Determine dose threshold correlated with aPTT unresponsiveness
Kartika et al, 2024	Retrospective cohort study	Single center	Tertiary medical center in the USA	Adult	95	VV	ARDS, severe COVID-19 infection	IV bivalirudin vs. IV heparin	• Duration of ECMO<br>• In-hospital LOS<br>• Need for CRRT<br>• ECMO days free of major bleeding (defined by ELSO)<br>• Thrombotic events: venous & arterial thromboembolism, in-circuit<br>• Survival to decannulation & discharge<br>• Number of blood products transfused (units)<br>• aPTT therapeutic range
Lopez et al, 2024	Retrospective chart review from 2016 to 2020	Single center	Tertiary academic medical center	Adults (+18 y.o.)	24	VV & VA	CS, respiratory failure	Bivalirudin monitoring<br><br>(HIT patients)	• Weight-based bivalirudin dose to therapeutic aPTT<br>• TTR (# of hours in goal aPTT range/ total duration on bivalirudin)<br>• Major bleeding: fatal or symptomatic in critical area or organ; or as defined by ELSO guidelines

									• Minor bleeding: acute or subacute overt bleeding, change in antithrombotic therapy, or discontinuation of anticoagulant<br>• Thrombotic events: DVT, PE, or circuit
Martin et al, 2024	Retrospective cohort study from January 2016 and October 2020	Single center	Tertiary medical center, cardiovascular ICU	Adult	68	Not specified	Acute myocardial infarction (AMI) or non-AMI indications	Cangrelor + bivalirudin +/- aspirin or Cangrelor + UFH +/- aspirin vs Bivalirudin or UFH +/- aspirin	• Incidence of major bleeding defined by ELSO criteria
Ogus et al, 2024	Retrospective cohort study from April 2020 - January 2022	Single center	ICU in Koşuyolu High Specialization Training and Research Hospital, Istanbul, Turkey	Adult	52	VV	CARDS	IV Bivalirudin + 150 mg of daily aspirin	• Dosing adjustments to aPTT & ACT values<br>• Major bleeding: ELSO criteria or bronchial, hematuria, intrathoracic or ICH<br>• Thromboembolic events: patient or circuit, ischemia, cerebrovascular, venous<br>• ICU LOS<br>• ECMO duration<br>• In-hospital mortality
Baldetti et al, 2023	Retrospective review from November 2019 to December 2021	Single center	ICU	Adults	14	VA	ACS- related CS/CA undergoing Percutaneous coronary intervention	Bivalirudin + cangrelor (starting dose: 0.125 μg/kg/min)	• Thrombotic events: arterial, ischemic, cerebellar stroke<br>• Major bleeding: BARC 3a, 3b, 5b<br>• In-hospital mortality
Buckley et al, 2023	Retrospective cohort over 2-year period	multicenter	Two academic university medical	Adult	104	Not specified	Critically ill	Bivalirudin – monitoring	• Determine the correlation coefficient between the standard TEG reaction (R)

			centers (Phoenix and Tucson, AZ, USA)						time & bivalirudin aPTT serum concentrations
Diaz et al, 2023	Retrospective observational cohort from February 2020 to November 2021	Single center	Mount Sinai Medical Center in Miami Beach, Florida, USA	Adults (+18 y.o.)	22	VV	COVID-19 infection	Bivalirudin vs. heparin	• Cannula duration<br>• Dosing & monitoring: time to aPTT & %TTR<br>• Thrombotic events: requiring circuit oxygenator exchange or detected by CT or ultrasonography<br>• Major bleeding incidences defined by ELSO guidelines<br>• Overall LOS<br>• In-hospital mortality<br>• Adverse drug reactions<br>• Weaning of ECMO
Hamzah et al, 2023	Retrospective study, matched 2:1 by age & ECMO type from June 2017 to May 2020	Multicenter	PICU	Pediatric (< 18 y.o.)	225	VA & VV	-	Bivalirudin vs heparin	• Odds of bleeding & thrombotic events<br>• Odds of transfusion<br>• In hospital mortality<br>• Neurological outcomes
Kaushik et al, 2023	Retrospective Comparative Cohort<br><br>(from January 2016 to December 2019)	Single center	PICU	Pediatric (<18)	40	VV & VA	Respiratory, cardiac, ECPR	Bivalirudin vs heparin	• ECMO duration<br>• Dose range & aPTT levels<br>• Minor & major bleeding events<br>• Hemolysis<br>• Mortality
Seelhammer et al, 2023	Retrospective cohort study	Single center	Large academic ECMO center	Adults	401	Not specified	-	Bivalirudin Vs heparin	• Holistic cost: wholesale acquisition & laboratory assays costs

Tong et al, 2023	Retrospective case-control study from February 2018 to February 2022	Single center	ICU	Adult	34	VV	-	Bivalirudin Vs. heparin	• ECMO duration<br>• Blood product transfusion<br>• Incidence of thrombosis & bleeding<br>• Weaning of ECMO<br>• In-hospital mortality
Uricchio et al, 2023	Retrospective cohort study from January 2009 to February 2021	Single center	Academic medical center	(18 y.o.+) Adults	143	VA	CS	Bivalirudin Vs. heparin	• ECMO-associated thrombotic events<br>• Major bleeding per ELSO criteria<br>• Blood product transfused<br>• Median ECMO duration
Bissell et al, 2022	Retrospective chart review: 9-month period	Single center	ICU	Adult	33	Not specified	SARS-CoV-2 associated acute respiratory failure	Bivalirudin Dose: 0.2 or 0.3 mg/kg/h<br><br>aPTT monitoring	• Dose adjustments<br>• Average TTR<br>• Rates of bleeding & venous thromboembolic events<br>• ECMO Survival<br>• ICU mortality rate
Hamzah et al, 2022	Retrospective analysis	Multicenter	PICU	Pediatric	75	Not specified	-	Bivalirudin + CRRT	• Estimate glomerular filtration rate<br>• Bivalirudin dose to therapeutic anticoagulation<br>• Correlate of dosage & severity of renal dysfunction
Hanna et al, 2022	Retrospective chart review	Single center	ICU, Cleveland Clinic Main Campus, Ohio, USA	HIT patients	12	VA & VV	-	Bivalirudin	• ECMO duration<br>• In-circuit thrombosis, DVT events<br>• Major bleeding

Lahart et al, 2022	Retrospective cohort from January 2019 to May 2020	-	PICU	Children (<18 y.o)	31	Not specified	-	Bivalirudin +/- CRRT	• CRRT, weight, or age affected Bivalirudin dosing<br>• ECMO duration
Pieri et al, 2022	Observational study from February 2020 to May 2020	Single center	ICU at a university hospital<br><br>(Milan, Italy)	Adults	83	VV & VA	Critically ill patients with SARS-CoV-2	Bivalirudin (IV infusion: 0.025 mg/kg/h) vs heparin (initial dose: 400 U/h)	• In-hospital & ICU mortality<br>• Thromboembolic complications: ischemic stroke, peripheral ischemia, PE, or other<br>• Bleeding events (GUSTO criteria)<br>• ICU & hospital LOS (days)<br>• Transfusion requirements
Rabinowitz et al, 2022	Retrospective review from 2018 to 2020	Single center	PICU	Pediatric	67 runs<br><br>1,500 lab values	Not specified	-	Bivalirudin vs heparin monitoring	• Correlation between bivalirudin & heparin dosing with PTT, TEG R-time, antifactor Xa (aXa) monitoring
Saeed et al, 2022	Retrospective from March 2020 to April 2021	Multicenter (17)	ICU	Adults	411	Not specified	COVID-19 infection	DTI (Bivalirudin/ argatroban) vs IDT (UFH)	• At 90 days in-hospital mortality<br>• DVT, ischemic & hemorrhagic stroke<br>• Bleeding requiring transfusion
Stammers et al, 2022	Prospective cohort data analysis from May 2020 to December 2021	Multicenter (47)	25 states in USA	Adults	570	VV	CARDS	Bivalirudin/Argatroban vs heparin	• Circuit failure change (oxygenator failure, circuit clots, pump malfunction, circuit hemolysis)<br>• Organ failure (hepatic, renal)<br>• Mortality<br>• ECMO duration (hrs.)

Teruya et al, 2022	Clinical, in-vitro	-	PICU, laboratory	Pediatric	17<br><br>(51 specimens)	Not specified	-	Bivalirudin Monitoring	• Compare aPTT, aPTT + heparinase (HPTT), dTT1:4, modified dTT1:10, ecarin chromogenic assay (ECA)
Trigonis et al, 2022	Retrospective case-control study from June 2019 to June 2020	Single center	Indiana University Health Methodist Hospital in Indianapolis, Indiana, USA	Adults	42	VV	ARDS secondary to COVID-19 or ARDS non-COVID etiology	Bivalirudin	• Time to goal aPTT<br>• % time at goal aPTT<br>• ECMO duration (hrs)<br>• In-hospital & ECMO mortality<br>• Total cost of bivalirudin<br>• ICU & hospital LOS<br>• Complications: ICH, GI, DVT
Campbell et al, 2021	Retrospective review	Single center	Tertiary care pediatric hospital	Pediatric	34	Not specified	-	Bivalirudin	• Average, initial & max doses<br>• Time to aPTT & time within goal aPTT range<br>• Bleeding & clotting complications<br>• Cost
Machado et al, 2021	Retrospective comparative study from September 2015 and January 2018	Single center	Pediatric cardiac ICU	Children (<18 y.o.)	32	VV & VA	Critically ill, PCICU	Bivalirudin vs heparin	• Average % time to aPTT & ACT<br>• Blood loss per hour<br>• Bleeding events<br>• Blood product transfusions (pRBCs)<br>• Survival to 6 months & decannulation
Rivosechhi et al, 2021	Retrospective observational cohort study from 2013 to 2020	Single center	Cardiothoracic ICU	-	295	VV	Respiratory failure	IV Bivalirudin vs IV heparin	• In-circuit thrombotic complications<br>• Time to circuit thrombosis<br>• Transfusion requirements<br>• Major bleeding events

Seelhammer et al, 2021	Retrospective chart review from January 2014 to October 2019	Single center	Large, high-volume tertiary referral ECMO center	Adult & Pediatric	424	VV & VA	Post-cardiotomy	Bivalirudin vs heparin	• Occurrence of HIT<br>• Mortality<br>• Transfusion requirements first 24 hrs.<br>• Labs per day<br>• Dose adjustments<br>• Ischemic complications
Schill et al, 2021	Retrospective comparative review study from June 2018 to December 2019)	Single center	Cardiac or pediatric ICU	Children: neonates to 18 y.o.	54	VV & VA	-	Bivalirudin vs heparin	• # of circuit interventions<br>• ECMO duration (days)<br>• Blood product transfusions<br>• Survival to decannulation & discharge
Hamzah et al, 2020	Retrospective chart review from October 2014 to May 2018	Single center	Tertiary, academic PICU	Pediatric	32	Not specified	-	Bivalirudin vs heparin	• Recovery<br>• Time to goal therapeutic anticoagulation level<br>• Bleeding events<br>• Rate of thrombotic events<br>• Comprehensive cost analysis
Kaseer et al, 2020	Retrospective observational study from January 2013 to September 2018	-	ICU	Adults	52	Not specified	-	Bivalirudin vs. heparin	• 7-day rate of composite thrombosis<br>• Major bleeding<br>• 30-day or in-hospital mortality<br>• % TTR of aPTT
Letunica et al, 2020	Age-specific retrospective comparative study	-	In-vitro, clinical laboratory	Healthy neonates, children & adults	120	-	-	Bivalirudin	• Thrombin inhibition by age: pharmacokinetic response / profile
Ryerson et al, 2020	Prospective observational	-	Medical-surgical &	Neonatal & Pediatric	18	VA & VV	Underlying cardiac diagnosis	Bivalirudin (0.16 mg/kg/h & titrated	• Circuit thrombosis

			cardiac PICUs		(20 runs)			in 10-20% increments) vs heparin	• Dosing (initial bolus, median range)<br>• ECMO duration (hrs)<br>• Circuit intervention<br>• Dose correlation with PTT, INR & ACT<br>• Bleeding complications<br>• Survival to hospital discharge
Snyder et al, 2020	Retrospective cohort from June 2016 to 2018	Single center	Peri-operative period	Neonates (less than or equal to 28 days)	42	VA	CDH- repair on ECMO	Bivalirudin monitoring	• Infusion rates & lab results<br>• # of transfusions<br>• Cannulation to repair<br>• Time to target aPTT & TEG-R levels
Teruya et al, 2020	Retrospective study from January 2017 to June 2018	Single center	Tertiary care pediatric hospital	Children	18<br><br>(106 blood sampl es)	ECMO or VAD	-	Bivalirudin monitoring	• Correlation between several ROTEM assays with max clot firmness & clotting time vs routine testing (aPTT, aPTT Hepzyme (HPTT), PT), fibrinogen, and platelet counts
Macielak et al, 2019	Retrospective 5-year study	Single center	ICU	Adults	153	VV & VA	-	Heparin vs Bivalirudin vs Heparin to bivalirudin	• Thromboembolic events per day<br>• Bleeding complications<br>• % measured aPTT in goal range
Walker et al, 2019	Retrospective Analysis	-	ICU	Adult	14	Not specified	-	Bivalirudin initial dose: 0.15 mg/kg/h	• Dosing adjustments to goal time in aPTT & due to RRT<br>• ECMO duration (days)<br>• Circuit change: thrombosis or failing oxygenation

									• Bleeding events: requiring reduction in aPTT goal range or anticoagulation cessation
Berei et al, 2018	Retrospective study	-	ICU	Adults	-	Not specified	-	Bivalirudin vs heparin	• Thrombotic events<br>• In-hospital & 30-day mortality<br>• % TTR<br>• Neurologic events<br>• Vascular & bleeding complications
**DTI: Argatroban**									
Burša et al, 2025	Observational study from April 2020 to July 2023	Single center	Intensive Care Department<br><br>(University Hospital Ostrava, Czech Republic)	Adults	63	VV	COVID-19 associated ARDS	Initial bolus UFH: 50-70 U/kg + 5000 U<br><br>Argatroban (5-10 ug/kg/hr.) vs. heparin (5-10 U/kg/hr.)	• ECMO duration (days)<br>• In-hospital mortality<br>• Weaning of ECMO<br>• Transfusion requirements<br>• Mortality-related bleeding (profuse bleeding with hemodynamic failure, leading to death) or life-threatening bleeding (6+ blood transfusions)
Burša et al, 2025	Prospective observational from April 2020 to July 2023<br><br>CTI:NCT06038682	Single center	Intensive Care unit / department<br><br>(University Hospital Ostrava, Czech Republic)	Adults	44	VV	Hospitalized with SARS-CoV-2	Initial UFH bolus: 50-70 U/kg IV + 5000 U<br><br>Argatroban: 0.3 ug/kg/hr<br>aPTT target: 40 – 60s<br>Anti-IIa target: 0.4 – 0.6 ug/mL	• Dose adjustments to Anti-IIa level<br>• State of circuit & flow rate<br>• Bleeding & thrombotic events<br>• In-hospital mortality

Mayerhöfer et al, 2024	Exploratory bi-centric cohort study from December 2019 to May 2021	Multicenter	Two tertiary care referral centers<br><br>(Medical University of Vienna & Medical University of Innsbruck)	Adults (+18 y.o)	57	VV	Severe CARDS	Initial UFH bolus: 5,000 – 10,000 IU<br><br>Argatroban infusion of 0.5 ug/kg/min vs Heparin bolus of 50 IU/kg bolus<br><br>Hemoclot level: 0.4 – 0.6 ug/mL	• Bleeding complications (BARC classification)<br>• Thromboembolism (PE, DVT, suspected or proven oxygenator clotting)<br>• Proportion of anticoagulation targets within range (aPTT, PT, thrombin time, dTT, Anti-Xa<br>• Mortality
Schiavnoi et al, 2024	Retrospective study from November 2020 to July 2021	single center	ICU<br><br>(Fondazione Policlinico Universitario Campus Bio-Medico in Rome, Italy)	Adults with heparin-associated thrombocytopenia (UFH)	40	VV	COVID-19-related bilateral interstitial pneumonia, acute hypoxic respiratory failure	Argatroban vs heparin	• Major & minor bleeding complications per ELSO guidelines (ICH, pulmonary, gastroenteric, intermuscular, respiratory system, venous cannulation areas)<br>• Platelet count, PT, aPTT, D-dimers
Menninger et al, 2023	Retrospective cohort study from January 2019 to February 2021	Single center	Department of Anesthesiology and Intensive Care Medicine<br><br>(University Hospital of Tuebingen, Germany)	Adults	57	VV	Severe ARDS	Argatroban vs. heparin<br><br>aPTT target: 50 – 60 s	• Days per oxygenator (# of oxygenators used per treatment period)<br>• Transfusion requirements<br>• Bleeding complications: ICH, GI, pulmonary, puncture sites, epistaxis, other<br>• Survival at 28 days
Cho et al, 2021	Retrospective nonrandomized study from	Single center	Community hospital	Adult	69	VV & VA	-	Argatroban: infusion of 0.5 mcg/kg/min vs	• Total cost of therapy<br>• Major bleeding events

	January 2017 to June 2018		(Georgia, USA)					heparin: infusion of 10 units/kg/h	• Circuit clotting, DVT, PE, ischemic events<br>• TTR & % of therapeutic PTTs<br>• Mortality
Fisser et al, 2021	Observational non-inferiority propensity scores matched study form January 2006 to March 2019	Single center	ICU at university hospital<br><br>(University Hospital Regensburg Bavaria, Germany)	Adults	117	VV	Severe respiratory failure or refractory respiratory acidosis	Argatroban vs heparin<br><br>aPTT was 50 +/- 5 sec	• Major & minor bleeding per ELSO criteria<br>• Major thrombosis (obstruction of >50%) & minor (obstruction < 50%)<br>• Transfusion requirements<br>• Technical complications: circuit thrombosis in oxygenator, pump head, oxygenator exchange<br>• Direct costs of drugs, blood products & HIT diagnostics
Dingman et al, 2020	Retrospective Cohort from January 2013 to June 2018	Single center	Surgical, medical, cardiac, cardiovascular ICU	Adults	80	Not specified	Critically ill	Argatroban: ECMO + CRRT or neither	• Organ dysfunction (modified SOFA)<br>• First therapeutic dose & adjustments for goal aPTT range<br>• Supratherapeutic aPTTs
Menk et al, 2017	Retrospective observational control-matched analysis from January 2007 to December 2014	single ARDS referral center	ICU department of Anesthesiology & Intensive Care Medicine<br><br>(Charité-	Adults	78	VV, pumpless ECLA (pECLA), VV-pECLA	Severe ARDS, organ failure	Argatroban vs UFH (control)	• Dosing & adjustments<br>• In-hospital mortality<br>• Major bleeding: ICH, pulmonary, retroperitoneal, or GI<br>• Minor bleeding: puncture site, nasopharyngeal zone, epistaxis, or skin lesions

			Universitäts medizin Berlin, Germany)						• Thromboembolic events: clotting in oxygenator, pump, cannulas, DVT, PE, limb ischemia, occlusive stroke<br>• Transfusion requirements<br>• Time to therapeutic aPTT values
**Serine Protease Inhibitor: Nafamostat Mesilate**									
Lee et al, 2022	Retrospective review from January 2017 to June 2020	Single center	Peri-operative setting<br><br>Haeundae Paik Hospital, Busan, South Korea	Adults	16	VA	Cardiovascular, respiratory & gastrointestinal disease	NM (rate: 0.2 – 0.5 mg/kg/hr) vs UFH with CRRT<br><br>aPTT range: 60 – 90 s	• Bleeding & thrombotic events<br>• ECMO duration (days)<br>• Median flows & gas exchange (range)<br>• Median infusion rate & dose of NM<br>• Time to & at laboratory tests (e.g., aPTT)<br>• RBC transfusions<br>• Adverse events
Han et al, 2019	Retrospective observational case series from January 2011 to December 2017	Single center	Chungnam National University Hospital, South Korea	Adults	87<br><br>(91 runs)	VV & VA	Respiratory, cardiac, post-cardiotomy, ECPR	NM infusion of: (0.5 mg/kg) or UFH infusion of: (7.5–20.0 units/kg/h)<br><br>Monitored: ACT (150 to 200s) & aPTT (55 to 70s)	• Bleeding episodes: cannulation or surgical site requiring intervention, cerebral hemorrhage, GI & airway bleeding<br>• Thrombotic episodes: intracardiac thrombus or circuit thrombosis, embolic stroke

Lim et al, 2016	Retrospective propensity scores matched review from January 2005 to November 2014	Single center	Department of thoracic & cardiovascular surgery, University of Ulsan College of Medicine, Asan Medical Center Seoul, Korea	Adults	320	VA	post-cardiotomy, shock, ECPR	NM (500 mg + dextrose 5%: rate of 20 mg/hr) vs<br><br>UFH (20,000 units + saline 500 mL: rate 800 units/hr)<br><br>ACT: 160 – 200s<br>aPTT: 50 – 70s	• Bleeding events: any bleeding requiring surgical intervention, cerebral hemorrhage, & GI bleeding<br>• Thrombotic events: embolic stroke, intracardiac thrombus<br>• ECMO duration (days)<br>• ECMO weaning
**Other**									
Buchtele et al, 2022	Randomized, double blind, placebo-controlled phase II pilot trial from September 2016 to April 2021	Multicenter	Two medical university<br><br>(Vienna, Austria)	Adults	48	VV	COVID-19 associated	Prostaglandin E1 (alprostadil):<br><br>PGE1 (IV: 5 ng/kg/min) vs Placebo (0.9% saline) + UFH	• Bleeding rate: # of RBCs transfused / study day<br>• Incidence of & time to clinically overt bleeding (Type II or higher according to BARC) & clotting events (PE, DVT)<br>• Oxygen transfer & transmembrane pressure<br>• Hemodynamic changes<br>• 90-day mortality
Buchtele, 2021	Prospective cohort study from January 2018 to February 2020	Single-center	Three medical ICUs<br><br>(Medical University of Vienna, Austria)	Adult	51<br><br>(117 samples)	VV & VA	-	Factor XII	• Change in Factor XII activity<br>• Prevalence of reduced coagulation Factor XII activity (<60%)<br>• Association of Factor XII activity with thromboembolic & bleeding events

Katz et al, 2021	Retrospective observational case series from June 2017 to September 2019	Single center	ICU	-	17	VA, Impella, or VA + Impella	CS post-percutaneous coronary intervention	Cangrelor (median dose of 0.75 µg/kg/min) + heparin + aspirin	• Cangrelor dose adjustments<br>• Bleeding events: major/minor, GI, peripheral cannula<br>• Thrombosis formation<br>• Survival
Yasuda et al, 2021	Retrospective observational cohort	Single center	ICU, Medical faculty of Oita University, Japan	Adults	15	VV	Severe respiratory failure	Recombinant human thrombomodulin (rhTM) vs rhTM + AT vs UFH + rhTM<br><br>All with CRRT	• ECMO duration (days)<br>• Median dose of rhTM, AT & UFH<br>• ACT & aPTT ranges<br>• Bleeding events: ICH & GI bleeding<br>• Thrombosis complications
Giani et al, 2020	Retrospective chart review from 2009 to 2018	-	ICU	Adult	48	VV	-	RCA+ UFH vs UFH alone<br><br>with CRRT	• Efficacy & safety for CRRT circuit<br>• Filter & circuit life span<br>• Rate of CRRT circuit clotting

Table S2. Anticoagulation-free or anticoagulation-sparing and biocompatibility strategies, presented by category and arranged in chronological order by author and year. The number of included studies per category is as follows: anticoagulation-free or anticoagulation-sparing (n = 5), and biocompatibility or circuit-modifying strategies (n = 1).

Author, year	Study Design	Single vs multicenter	Setting	Population	Size (n)	ECMO Modality	Indication for ECMO	Intervention or Comparator	Outcome measures
**Anticoagulation-free or sparing**									
Kurihara et al, 2025	Retrospective cohort from January 2023 to April 2025	Single center	EMR<br><br>(Northwestern University Medical	Adults	7	VA	Bi-lateral re-do lung transplantation	5,000 U of UFH before cannulation<br><br>Cannulas - not	• Intraoperative outcomes<br>• Transfusion requirements<br>• ECMO duration

			Center in Chicago, Illinois, USA)					heparin coated<br><br>Tubing & oxygenator - heparin coated	• Post-operative complications<br>• Survival<br>• Incidence of acute kidney injury/ primary graft dysfunction<br>• Thromboembolic events: DVT & PE<br>• ICU & overall hospital LOS
Qi et al, 2024	Retrospective cohort from August 2017 & July 2022	Single center	Early perioperative period<br><br>(China-Japan Friendship Hospital, Beijing, China)	Adults	324	VV, VA & VV-A	Bridge to lung transplantation	No anticoagulant administered 24 hr before surgery<br><br>Sodium citrate anticoagulant to those requiring RRT<br><br>Heparin-free: no heparin or heparin derivatives used during, 3 days after, or during ECMO run period	• ECMO duration<br>• Bleeding events: intraoperative bleeding, thoracic drainage within 24 hrs post-op, ICH, GI, puncture site bleeding<br>• Thrombotic events: limb necrosis, AMI, acute cerebral infarction, PE, mesenteric arteriovenous<br>• Transfusion of blood products<br>• Mortality<br>• Prognostic factors of 1-year survival
Zhao et al, 2022	Retrospective study from June 2021 to September 2020	Single center	First Affiliated Hospital of Zhegzhou Univesity, Henan, China	(18 y.o +)	70	VV	Trauma, post-surgery	Low UFH bolus: 3000 IU w/o subsequent infusion (Heparin-free)	• 30-day ICU mortality<br>• Thromboembolic events: lower extremity, membrane lung, circuit, cannula<br>• Severe bleeding: need for

									intervention or >10 RBC transfusions<br>• Oxygenator change
Wood et al, 2020	Retrospective cohort review from May 2011 to January 2018	Single center	ICU	Adults	203	VA	-	No systemic anticoagulation vs systemic anticoagulation	• Hemorrhagic & thrombotic complications<br>• Blood product use<br>• Hospital LOS<br>• In-hospital mortality
Carter et al, 2019	Comparative retrospective study from October 2011 to May 2018	Single center	Cardiothoracic ICU	Adult	40	VV	Trauma	Heparin sparing:<br><br>P.h.i.s.i.o-coated polyvinylchloride tubing + oxygenators & pump heads none heparin-coated + no systemic heparin	• Duration of ECMO<br>• ICU & hospital LOS<br>• Renal failure (CRRT)<br>• Transfusion requirements<br>• Survival to decannulation & discharge
**Biocompatibility**									
Moussa et al, 2025	Retrospective propensity score weighted clinical cohort analysis (from 2013 to 2020)<br><br>Prospective histologic cohort (WITECMO-H multicenter study: NCT03070912)	Multicenter	3 ICU academic ECMO referral centers<br><br>(France)	Adult	461	VA	Severely ill, CS	PPC vs Heparin coating	• Clinical endpoints: Thrombotic & bleeding complications Mortality<br>• Histological Analysis of clots: Content of vWF, platelets, fibrinogen, neutrophil extracellular traps

Table S3. Monitoring and modeling strategies, arranged in chronological order by author and year. The number of included studies is (n = 6).

Author, year	Study Design	Single vs multicenter	Setting	Population	Size (n)	ECMO Modality	Indication for ECMO	Intervention or Comparator	Outcome measures

Malik et al, 2026	Retrospective cohort study from 2006 to 2021	Single center	Canada	Adult	109	-	-	Joint longitudinal - survival modeling framework: evaluating associations between aPTT trajectories & adverse outcomes	• Time to first major bleeding event (BARC ≥3); death being a competing risk
Brinkley et al, 2025	Quantitative methods modeling	-	-	Pediatric / Children	-	-	-	Use baseline data (patient information, bivalirudin doses, PTT measures) to predict optimal therapeutic bivalirudin dose	• Compare the modeled predicted dose & actual starting dose<br>• Determine the best predictors of therapeutic bivalirudin dose
Frydman et al, 2022	Retrospective laboratory sample analysis & chart review	Single site	105-bed pediatric & cardiac ICUs Texas Children's Hospital & Baylor College of Medicine Houston, TX	Pediatric	15<br><br>(41 specimen)	VV & VA	-	Bivalirudin vs heparin<br><br>CTS monitoring	• The ability of Factor IIa Clotting Time Score (CTS) to detect & quantify the anticoagulation effect
Giani et al, 2021	Prospective observational study	Single center	ICU	Adult	25	VV & VA	-	UFH with ROTEM monitoring	• Evaluate ROTEM & compare it to TEG & conventional assays (aPTT, TEG R time, ROTEM (INTEM CT)
Panigada et al, 2018	Prospective, randomized, controlled pilot trial	Multicenter	Two academic tertiary care centers<br><br>(Milan & Palmero, Italy)	Adults (18 y.o +)	42	VV	ARDS or as bridge to lung transplant	UFH with TEG-based protocol vs standard of care aPTT-based protocol	• # of hemorrhagic (BARC scores) or thrombotic events<br>• Transfusion requirements<br>• # of heparin dose adjustments, R-K

									or aPTT ratio within target range<br>• # of circuit failures/changes<br>• Correlation between heparin dose & aPTT & R-K time<br>• Cost-analysis
Henderson et al, 2018	Retrospective chart review over 2-year period from July 2013 to July 2015	Single center	Large tertiary care center, freestanding, children's hospital medical & cardiac ICU	Pediatric & neonate<br><br>(<18 y.o. & +2kg)	30	VV & VA	Congenital heart disease, CA, CS	UFH with TEG monitoring	• Correlation between TEG & conventional measures in predicting thrombotic & bleeding events (defined by ELSO guidelines)<br>• Determine optimum values for TEG R time (TEG RCK) & anti-Xa activity to prevent thrombosis & bleeding events

Table S4. Identified clinical trials organized by country in alphabetical order; countries with multiple trials are further arranged by trial start year (n = 22)

Clinical Trial: Title & Registration ID	Location	Funding Sponsor	Study Design	Size	Age group	Single vs multicenter	Outcomes	Strategy
Bivalirudin versus Heparin in Extracorporeal Membrane Oxygenation (BLUSH)<br><br>(ClinicalTrials.gov: NCT05959252/2023/ETH00443) Started: 2024	New South Wales, Australia	Sydney Local Health District	Interventional, open label randomized clinical trial<br><br>Phase 2b-recruiting	80	Adults	Single center	• TTR, mean aPTT & anti-Xa<br>• Circuit changes<br>• Serious adverse events<br>• Thrombotic events (# of DVT), major bleeding events (defined by ISTH) & bleeding events defined by BARC<br>• Survival to ICU & hospital discharge	Bivalirudin vs UFH

							• Blood product usage<br>• Cost	
Comparison of three different blood-thinners for the use during therapy with the heart-lung life-support machine<br><br>ClinicalTrials.gov & EU CTR: NCT06442267/1644/2022-002259-20, started: 2024	Vienna, Austria	Medical University of Vienna	Interventional, three-arm randomized controlled non-inferiority pilot study<br><br>Phase 4 - recruiting	90	Adults	-	• Incidence of thromboembolic events<br>• Bleeding events defined by BARC<br>• Frequency of transfusions<br>• LOS in ICU<br>• Mortality	Argatroban vs Enoxaparin vs UFH
Safety and Feasibility of Argatroban as Anticoagulant in Adults with ECMO<br><br>Clinicaltrials.gov: (NCT05226442) Started: 2021	Vienna, Austria	Medical University of Vienna	Interventional, prospective, randomized, controlled pilot<br><br>Phase 2/3 - completed	40	18+ (VV & VA)	Single center	• Bleeding (per BARC criteria) & thrombosis events (PE, DVT)<br>• Transfusion requirements<br>• Mortality	Argatroban vs UFH
A prospective randomized pilot trial on safety and feasibility of Argatroban as blood thinner in patients with heart-lung machine<br><br>WHO ICTR: (EUCTR2021-001456-34-AT) Started: 2021	Vienna, Austria	Medical University of Vienna	Prospective randomized pilot trial<br><br>Phase 2 - on going	40	Adults	Single center	• Safety & feasibility<br>• Bleeding events: # of pRBCs transfused<br>• Venous thrombosis<br>• Global clotting tests & Hemclot assay, TEG	Argatroban vs UFH
A prospective randomized, double blind study on safety and efficacy of Alprostadil as additional Anticoagulant in Patients with veno-venous ECMO<br><br>ID: (EUCTR2015-005014-30-AT) Started: 2016	Vienna, Austria	Medical University of Vienna	Interventional, prospective, randomized, double blind<br><br>Completed	60	Adults (VV)	-	• Measure efficacy as an additional anticoagulant<br>• Reduction in bleeding rate: need of pRBCs	PGE: Alprostadil + UFH
PGE1 as Additive Anticoagulant in ECMO-	Vienna, Austria	Thomas Staudinger	Interventional, randomized,	50	18+ (VV)	Single center	• Bleeding rate: # of pRBCs transfused	PGE1 (Alprostadil) vs placebo (sodium

Therapy (ECMO_PGE1)<br><br>ClinicalTrials.Gov: (NCT02895373) Started: 2016			double-blind, placebo-controlled trial<br><br>Phase II - Terminated				• # of bleeding incidences, clotting events, oxygenator changes<br>• Global coagulation assays & TEG<br>• Mortality<br>• # of transfusions	chloride solution) + UFH
Thrombo-Elastography Guided Management of ECMO (TEGMO)<br><br>ClinicalTrials.gov: (NCT04268940) Started: 2020	Toronto, Ontario, Canada	Damian Ratano	Observational prospective cohort<br><br>Unknown Status	61	18+	Single center	• Correlation of TEG & aPTT<br>• Correlation of TEG parameters with classical coagulation tests<br>• TEG's ability to identify patients at higher risk of bleeding or clotting	TEG6s + UFH
Anticoagulation Free VV-ECMO for Acute Respiratory Failure<br><br>ClinicalTrials.gov: (NCT04273607) Started: 2022	Toronto, Ontario, Canada	Damian Ratano	Interventional, randomized, parallel assignment<br><br>Phase 2/3 - unknown status	40	Adult (VV)	Single center	• Associated thrombotic complications<br>• Hemorrhagic complications per BARC<br>• Transfusion requirements	Anticoagulation-free: subcutaneous enoxaparin or UFH
Use of Bivalirudin for Anticoagulation in Patients with Extracorporeal Membrane Oxygenation<br><br>ClinicalTrials.gov:NCT06275555/2023-101, started: 2024	Beijing, China	Xiaotong Hou	Interventional, randomized, controlled trial<br><br>Unknown status - recruiting	154	Adult<br><br>(VA & VV)	Single center	• Thrombotic & bleeding complications<br>• Mortality<br>• Transfusions<br>• Time to & at target aPTT	Bivalirudin vs UFH
Use of Nafamostat Mesilate for Anticoagulation in Patients With ECMO<br><br>ClinicalTrials.gov: NCT06276010, started:2024	Beijing, China	Xiaotong Hou	Interventional - recruiting	80	18 + (VV & VA)	Single center	• Incidence of thrombotic events, severe bleeding per ELSO guidelines & oxygenator dysfunction<br>• Infusion volume of blood products<br>• Mortality<br>• Time to target anticoagulant level	NM vs UFH

A Novel Strategy of ECMO Management Using Nafamostat for Regional Combined With Low Intensity Systematic Anticoagulation<br><br>ClinicalTrials.gov:NCT06676085, started: 2024	Beijing, China	Beijing Chao Yang Hospital	Interventional, randomized parallel study<br><br>Recruiting	60	18+	Single center	• Incidence of bleeding or thrombotic events	NM vs UFH
Efficacy and Safety of Nafamostat Mesylate for VV-ECMO Anticoagulation<br><br>ClinicalTrials.gov:NCT05555641/NMST20211022, started: 2022	Wuhan, Hubei, China	Xiaobo Yang	Interventional, randomized, single-blind, multicenter exploratory, Heparin-controlled trial<br><br>Phase 2 - recruiting	40	Adults (VV)	Multicenter	• Incidence of severe bleeding, thrombosis<br>• Oxygenator replacement frequency<br>• Compliance rate of aPTT test<br>• Case fatality rate<br>• In-hospital mortality<br>• ICU & hospital LOS<br>• Transfusion requirements	NM vs UFH
Safety and Efficacy of Argatroban Applicated in Anticoagulation of V-V ECMO<br><br>Clinicaltrials.gov:NCT04925167, started: 2021	Beijing, China	Beijing ChaoYang Hospital	Interventional - completed	20	18+ (VV)	Single center	• Incidence of ECMO related thrombotic events	Argatroban vs UFH
Monitoring Anticoagulation in Patients on ECMO for Severe Lung Failure<br><br>ClinicalTrials.gov:NCT06038682/, started:2020	Ostrava, Czechia	University Hospital Ostrava	Observational - completed	100	Adults (VV)	Single center	• Compare anti-IIa & aPTT values with argatroban dose; compare APT & anti-Xa values with heparin dose<br>• Frequency of bleeding & thrombotic complications<br>• Blood products used	Argatroban vs heparin
Thrombin Generation as a New Test for Anticoagulation Management in Patients With ECMO	Strasbourg, France	University Hospital, Strasbourg, France	Observational - completed	30	18+ (VV & VA)	Single center	• Endogenous thrombin potential (ETP)<br>• Levels of markers of vascular cell activation	Thrombin generation blood test

Clinicaltrials.gov:NCT03832842, started: 2019							• Incidence of bleeding defined by WHO classification.<br>• Incidence of thrombotic & ischemic events: ischemic stroke, limb ischemia, myocardial infarction	
ECLS-Therapy in HIT II patients – safety of Argatroban- vs Heparin therapy: eCMO-SAvHE - Study<br><br>WHO ICTRP: DRKS00011416, started: 2018	Germany	Universitätsklinikum Gießen und Marburg	Retrospective Observational<br><br>Recruiting - on going	200	Adults (VV & VA)	Multicenter	• Non-sublimity of Argatroban therapy<br>• Bleeding & thrombus complications<br>• Mortality<br>• Frequency of filter changed	Argatroban vs UFH
Study the effect of heparin, bivalirudin in heart surgery in children less than 1 year<br><br>WHO ICTRP: CTRI/2024/11/077189, started: 2024	New Delhi, India	All India Institute of Medical science, New Delhi	Interventional, randomized parallel controlled trial<br><br>Phase 4 - recruiting	20	Neonates & infants (<1 year)<br><br>(ECMO-CPB)	-	• Safety & efficacy<br>• Determine appropriate dosage (during cardiac surgery & ICU)<br>• Postoperative blood loss<br>• Transfusion requirements<br>• Major bleeding & thromboembolic events<br>• ICU LOS<br>• Mortality	Bivalirudin Vs UFH
The efficacy of TEG6s as the management of anticoagulation therapy during pediatric ECMO<br><br>WHO ICTRP: JPRN-jRCT1012220032, started: 2022	Hakkaido, Japan	Sakai Wataru	Observational study & recruiting	34	Children <10 y.o.	-	• Brain or tracheal hemorrhage or circuit closing<br>• Mortality	Monitoring: TEG6s
Cangrelor on Top of anticoagulation in Patients With myocardial Infarction-related Cardiogenic Shock/Cardiac Arrest receiving VA-	Milan, Italy	IRCCS San Raffaele	Interventional<br><br>Phase 2 - recruiting	50	Adults (18+) (VA)	Single center	• Major bleeding per BARC classification<br>• Thrombotic events: MI, any arterial or venous thromboembolism, ischemic stroke	P2Y12 Inhibitor: Cangrelor

ECMO (SURVIVE)<br><br>ClinicalTrials.gov:NCT06792643, started: 2024							• Multiplate assay with ADP-test values	
Prospective Multicenter Observational Study on Transfusion Practice in Vv-ECMO Patients: The PROTECMO Study (PROTECMO)<br><br>ClinicalTrials.gov:NCT03815773, started: 2018	Palmero, Italy	The Mediterranean Institute for Transplantation and Advanced Specialized Therapies	Observational international, prospective observational cohort study<br><br>Completed	656	Adult<br><br>(VV)	Multicenter	• Descriptive data of transfusion requirements<br>• Descriptive data of anticoagulation practices	• Overall anticoagulation monitoring & strategies adopted per center
Growth of High-Quality Oxides on The Inner Surface of ECMO Circuit by ALD to Reduce Thrombus Formation<br><br>Clinicaltrials.gov: NCT03662594, started: 2018	Taipei, Taiwan	National Taiwan University Hospital	Interventional - completed	4	Adults (+20)	Single center	• Protein content or configuration changed	Biocompatibility: atomic layer deposition (ALD) tube
Safety and Efficacy of Bivalirudin Versus Heparin for Systemic Anticoagulation in Extracorporeal Membrane Oxygenation<br><br>ClinicalTrials.gov: (NCT03965208) Started: 2019	Portland, Oregon, USA	Legacy Health System	Interventional, open-label parallel group randomized pilot study<br><br>Phase 4 - completed	34	Adults (+18)	Single center	• % TTR<br>• Major bleeding & thrombotic events<br>• Duration of oxygenator<br>• Blood products used	Bivalirudin vs UFH

Table S5. Identified abstracts, poster sessions, and guidelines from 2020 to 2025, organized chronologically by author and year (n = 35).

Author, year	Conference, journal, abstract #	Location	Study design	Size (n)	Population	ECMO Modality	Strategy	Outcomes

Frazzei et al, 2025	ESICM LIVES 2025, Munich Germany<br><br>Intensive Care Medicine Experimental, 2025;13(1): 000672 https://doi.org/10.1186/s40635-025-00797-x	Italy	Prospective randomized crossover study	15	Adult ICU	VV + CRRT	RCA (4% sodium citrate) + UFH vs UFH alone<br><br>TEG monitoring	• Circuit clotting & lifespan<br>• Platelet, D-dimers, fibrinogen levels<br>• Adverse events
Senarighi et al, 2025	13th EuroELSO, 23-26 April 2025, Milan, Italy<br><br>#247, Perfusion, Vol 40, Issue1 https://doi.org/10.1177/02676591251327030	Italy	Observational study from January 2018 to January 2024 at a referral center	45	Adult	VA + Impella – CS	Bivalirudin + antiplatelet therapy<br><br>aPTT: 55-70 sec	• Support duration (days)<br>• Major bleeding event per BARC guideline<br>• Thrombotic complications<br>• In-hospital survival
Zhang et al, 2025	ESICM LIVES 2025, Munich, Germany<br><br>Intensive Care Medicine Experimental 2025, 13(1): 001125 https://doi.org/10.1186/s40635-025-00797-x	China	Multicenter retrospective study	1,542	-	-	Machine Learning modeling: sepsis-induced coagulopathy	• Identify distinct coagulation phenotypes using temperature trajectories<br>• Correlate of each phenotype with coagulopathy severity & mortality<br>• Phenotype correlation with therapeutic anticoagulation response
Zoppellet o et al, 2025	13th EuroELSO - 23-26 April 2025 - Milan, Italy<br><br>#301, Perfusion, Vol 40, Issue 1, pg. 67S-291S https://doi.org/10.1177/02676591251327030	Italy	Retrospective single-center cohort study at tertiary care center PICU from 2018 to 2024	40	Pediatric & neonatal	VA - arterial reconstruction or ligation	Acetylsalicylic acid, UFH, warfarin, bivalirudin,	• Survival to decannulation<br>• Decannulation strategy & post-decannulation anticoagulation<br>• Vascular patency<br>• Neurological outcomes
Altinay et al, 2024	EuroELSO 2024, 24th–27th April, Krakow, Poland<br><br>#310, Perfusion, Vol 39, Issue 1 https://doi.org/10.1177/02676591241239138	Turkey	Retrospective cohort review from April 2020 to February 2022	64	Adults	VV - COVID-19 respiratory failure	Bivalirudin	• ECMO duration<br>• Weaning off ECMO<br>• Major bleeding complications perioperative period

Johng et al, 2024	35th Annual ELSO Conference Abstracts, September/October 2024<br><br>ASAIO Journal 70, Suppl 4, p. 76, #235 10.1097/01.mat.0001070200.19953.68	Multiple	Multicenter anticoagulation survey, Children's Hospital Neonatal Consortium from April to May 2024	39	Neonatal	Not specified	Bivalirudin	• Frequency & Indications of use as primary anticoagulant<br>• Dosing<br>• Bleeding & clotting complications
Khoury et al, 2024	35th Annual ELSO Conference Abstracts, September/October 2024,<br><br>ASAIO Journal 70, Suppl 4, #198 10.1097/01.mat.0001070200.19953.68	-	Retrospective single-center investigation from January 2020 to May 2021	103	-	VV & VA	Bivalirudin monitoring: low & high aPTT levels	• Survival to decannulation & discharge<br>• Thrombotic complications: oxygenator failure, cannula, DVT/PE, cardioembolic stroke, limb arterial<br>• Bleeding complications: ICH, GI, airway, surgical or cannula site, epistaxis<br>• Average time on ECMO
López et al, 2024	Abstracts from EuroELSO 2024, 24th–27th April, Krakow, Poland,<br><br>#300 Perfusion, Vol. 39, Issue 1, https://doi.org/10.1177/02676591241239138	Spain	Observational retrospective cohort study from March 2020 to February 2022	62	ICU HIT patients	Not specified - COVID-19	Bivalirudin vs UFH	• Duration on ECMO<br>• Maximum flow<br>• Bleeding complications: major, hemothorax, lung bleeding<br>• Pulmonary embolism<br>• ICU length<br>• ICU survival at discharge
Ward et al, 2024	35th Annual ELSO Conference Abstracts, September/October 2024, #133<br><br>#133, ASAIO Journal 70, suppl 4, 10.1097/01.mat.0001070200.19953.68	Ohio, USA	Retrospective chart review from January 2015 to July 2021	114	Adult	VV - bridge to transplant	Systemic anticoagulation vs no-systemic anticoagulation	• Duration of ECMO (days)<br>• Incidence of oxygenator exchange<br>• Hemorrhagic & systemic thrombotic events
Bikdeli et al, 2023	American Heart Association's Vascular Discovery: From Genes	USA	Data from an open label, randomized controlled trial	562<br><br>(24 models)	-	-	Modeling using ITEs (individualized treatment effects)	• Accuracy in identifying patients deriving benefit or harm by intervention<br>• Accuracy in identifying any heterogeneous treatment effect

	to Medicine 2023 Scientific Sessions<br><br># 260, American Heart Association Journals, Vol 43, Supplement 1		(INSPIRATION)					
Daniel, et al, 2023	34th Annual ELSO Conference Abstracts, September/October 202, Seattle, Washington<br><br>#103, ASAIO Journal Vol 69, Suppl 3, ISSN 1058-2916	USA	Single-center retrospective study	68	Neonate	Not specified	Bivalirudin vs UFH	• Dollar cost per ECMO hour<br>• Incidence of ICH<br>• Survival rates<br>• Drug costs (wholesale price)
*Han et a*l, 2023	34th Annual ELSO Conference Abstracts, September/October 2023, Seattle, Washington<br><br>#122, ASAIO Journal, Vol 69, Suppl 3 ISSN 1058-2916	South Korea	Safety & feasibility: Level 1 Trauma center retrospective review from January 2016 to December 2022	34	Severe trauma patients	VV - respiratory distress	Systemic anticoagulation vs. anticoagulation-free	• ECMO duration time<br>• Mortality<br>• Hemorrhage & thromboembolic complications<br>• Emergency circuit change
Kirali et al, 2023	EuroELSO 2023, 26-29 April 2023, Lisbon, Portugal<br><br>#418, Perfusion, Vol 38, Issue 1, pgs. 82-212 https://doi.org/10.1177/02676591231162023	Turkey	Prospective study	68	Adults	VV - COVID ARDS	Bivalirudin	• Mean duration of ECMO<br>• Survival rate<br>• Hypoxic periods
Stark et al, 2023	34th Annual ELSO Conference Abstracts, September/October 2023, #194<br><br>#194, ASAIO Journal, Vol 69, Suppl 3 ISSN 1058-2916	USA	Retrospective analysis of CDH neonates from 2021 to 2023	12	Neonates	Not specified - CDH	Bivalirudin	• ICH events & bleeding complications requiring surgical intervention<br>• Survival to decannulation, 6 months of age & discharge<br>• Duration of ECMO (Days)
Vale et al, 2023	EuroELSO 2023, 26-29 April 2023, Lisbon, Portugal<br><br>#116, Perfusion, Vol 38, Issue 1, pgs. 82-212,	Australi a	Single-center retrospective case series in tertiary ECMO center	11	-	Not specified	Bivalirudin: aPTT monitoring	• ECMO duration<br>• Median dose & adjustments<br>• aPTT measurements<br>• Requirement for transfusions

	https://doi.org/10.1177/02676591231162023							• Major bleeding & thrombotic events
Altinay et al, 2022	10th EuroELSO Congress,4-6 May 2022 , London, UK,<br><br>#134, Perfusion, Vol 37, Issue 1, pgs. 3-111 https://doi.org/10.1177/02676591221089240	Turkey	Retrospective analysis from April 2021 to December 2021	11	Peripartum period	VV - COVID-19 ARDS	Bivalirudin	• Duration of ECMO<br>• Weaning of ECMO<br>• Survival to discharge
Dianderas et al, 2022	10th EuroELSO Congress, 4-6 May 2022, London, UK<br><br>#172, Perfusion, Vol 37, Issue 1, pgs. 3-111 https://doi.org/10.1177/02676591221089240	Spain	Prospective observational study from March 2020 to July 2021	49	-	Not specified - COVID-19 related ARDS	Bivalirudin vs UFH	• Dose adjustments<br>• Membrane oxygenator half-life
DiGeronimo et al, 2022	33rd Annual ELSO Conference Abstracts, September, Boston, Massachusetts, 2022 ASAIO Journal Vol. 68, Supp. 3, ISSN 1058-2916	USA	Cross-sectional survey of Level IV NICUs across CHNC from May to July 2022	22 centers	Neonatal	VV & VA	Bivalirudin	• Clinical ECMO indications<br>• Centrifugal pump type<br>• Anticoagulant indication<br>• Dose adjustments<br>• Incidence of bleeding<br>• Circuit life span<br>• Blood product transfusion requirements
Handley et al, 2022	33rd Annual ELSO Conference Abstracts, September, Boston, Massachusetts, 2022 ASAIO Journal Vol. 68, Supp. 3, ISSN 1058-2916	USA	Single-center retrospective review from August to November 2021	6	Neonatal	Not specified - respiratory failure	Bivalirudin	• ECMO duration<br>• Circuit changes<br>• Bleeding & hemorrhage complications<br>• Transfusion requirements<br>• Severe intracranial bleeding or stroke<br>• Survival to decannulation & hospital discharge
Hickman et al, 2022	33rd Annual ELSO Conference Abstracts, September, Boston, Massachusetts, 2022	USA	Prospective	17	-	VV & VA	Biocompatibility: AMG PMP & Medtronic Nautilus Smart (NautilusTM)	• Oxygen transfer & pressure drops<br>• Duration of support<br>• Circuit failure

	SAIO Journal Vol. 68, Supp. 3, ISSN 1058-2916							
Lerner et al, 2022	Supplement for the 10th EuroELSO Congress, 4-6 May, 2022, London, UK,<br><br>#185, Perfusion, Vol 37, Issue 1, pgs. 3-111 https://doi.org/10.1177/02676591221089240	Israel	Retrospective cohort at a single tertiary center from 2021 to 2021in critical care department	50	-	VV - respiratory failure	UFH or Bivalirudin	• Duration of ECMO<br>• Survival rates<br>• Thrombotic & bleeding events beetween COVID-19 & non-COVID 19 patients
Lines et al, 2022	33rd Annual ELSO Conference Abstracts, September, Boston, Massachusetts, 2022 ASAIO Journal Vol. 68, Supp. 3, ISSN 1058-2916	USA	Retrospective, matched, cohort	50	<18 y.o.	VV & VA	UFH vs Bivalirudin	• % time in TTR<br>• Major/minor bleeding events<br>• Thrombotic events<br>• Circuit exchange due to thrombosis<br>• Mortality<br>• Cost of AT3 supplementation
McMichael et al, 2022	2021 ELSO Adult and Pediatric Anticoagulation Guidelines ASAIO Journal 2022 DOI: 10.1097/MAT.0000000000001652	-	Anticoagulation Guidelines	-	Adults & pediatric	VV & VA	UFH, DTIs, recombinant FVIIa, & therapeutic monitoring	• Recognized anticoagulants, monitoring assays, and outcome definitions
McMillan et al, 2022	33rd Annual ELSO Conference Abstracts, September, Boston, Massachusetts, 2022 ASAIO Journal Vol. 68, Supp. 3, ISSN 1058-2916	USA	Retrospective review from June 2020 to June 2022	44	Pediatric	VV & VA - congenital heart disease	Bivalirudin, Argatroban, UFH	• Circuit changes<br>• Mean ECMO duration<br>• Mean time to therapeutic PTT<br>• Mean dose<br>• Thromboembolic events<br>• Survival to decannulation
Seelhammer et al, 2022	33rd Annual ELSO Conference Abstracts, September, Boston, Massachusetts, 2022 ASAIO Journal Vol. 68, Supp. 3, ISSN 1058-2916	USA	Single center retrospective cohort study from January 2015 to May 2021	338	Adult & Pediatric	VV & VA	Platelet Inhibitor (PI)	Association between PI &:<br>• Hazard for circuit interventions<br>• Hemolysis<br>• Thrombotic complications<br>• Blood product administration

Urbas et al, 2022	33rd Annual ELSO Conference Abstracts, September, Boston, Massachusetts, 2022 ASAIO Journal Vol. 68, Supp. 3, ISSN 1058-2916	USA	Retrospective cohort data at tertiary care hospital from February 2019 to December 2021	-	Neonates & Pediatric	VV & VA	Bivalirudin vs UFH	• CRRT administration<br>• Median aPTT, anti-Xa, PTT values<br>• Filter duration
Brozzi et al, 2021	32nd Annual ELSO Conference Abstracts, #142 ASAIO Journal, September-October 2021, Vol. 67, Supp. 3, ISSN 1058-2916	USA	Retrospective chart review March 2020 to June 2021	12	Adults	VV & VAV	Bivalirudin vs UFH<br><br>PTT goal: 50-70s	• Mean ECMO support (hrs)<br>• # of achieved therapeutic PTT range<br>• Bleeding complications<br>• Mortality
Danzo et al, 2021	32nd Annual ELSO Conference Abstracts, #93 ASAIO Journal, September-October 2021, Vol. 67, Supp. 3, ISSN 1058-2916	USA	Single-center retrospective review from 2018 to 2021	36	Pediatric	Not specified	Anti-coagulation Free	• Bleeding & thrombotic events (own definitions)<br>• Duration of ECMO<br>• Flow rates<br>• Survival to decannulation & discharge
Hamed et al, 2021	32nd Annual ELSO Conference Abstracts, #104 ASAIO Journal, September-October 2021, Vol. 67, Supp. 3, ISSN 1058-2916	USA	Retrospective chart review May 2020 to May 2021	31	Pediatric	Not specified	Bivalirudin vs UFH<br>+ low-flow or clamp trials	• ECMO flows<br>• Heparin boluses<br>• Bivalirudin infusion rates<br>• Anticoagulation profiles<br>• Blood product administration<br>• Circuit interventions
Lima et al, 2021	32nd Annual ELSO Conference Abstracts, #147 ASAIO Journal, September-October 2021, Vol. 67, Supp. 3, ISSN 1058-2916	USA	Retrospective chart review, single center, tertiary pediatric institution from June 2020 to March 2021	5	Children	VA - heart failure & arrhythmia	UFH vs DTI vs UFH + DTI	• Thrombus & hemorrhage complications<br>• Survival to hospital discharge<br>• Neurological outcomes<br>• Cardiac function
Brizzi et al, 2020	31st Annual ELSO Conference Abstracts, #191 ASAIO Journal, September 2020, Vol. 67, Supp. 3, ISSN 1058-2916	Italy	Retrospective analysis from March 21 to May 18 2020	3	-	VV & VAV - respiratory failure & CS	Monitoring: ROTEM (UFH infusion)	• ROTEM values correlation with a hypercoagulable state

Chandel et al, 2020	31st Annual ELSO Conference Abstracts, #181<br>ASAIO Journal, September 2020, Vol. 67, Supp. 3, ISSN 1058-2916	USA	Single-center analysis	24	COVID patients	Not specified	Monitoring: TEG (UFH vs Bivalirudin)	TEG value correlations with<br>• Hypercoagulable state<br>• Thrombotic events<br>• D-dimers<br>• Fibrinogen
Grady et al, 2020	31st Annual ELSO Conference Abstracts, #162<br>ASAIO Journal, September 2020, Vol. 67, Supp. 3, ISSN 1058-2916	USA	Single-center descriptive observational study from June 2020 to August 2020)	16	-	VV/hybrid/ VA-respiratory failure, post-cardiotomy, cardiac failure	Biocompatibility: PMP fiber Nautilus TM ECMO oxygenator	• Duration on ECMO<br>• Weaning of ECMO<br>• Mechanical complications<br>• Oxygenator changes<br>• Oxygenator gas exchange
Giuliano et al, 2020	EuroELSO 2020 Poster<br><br>Abstract # 308, Perfusion, Vol 35, Issue 1, suppl	USA	Retrospective analysis from January 2016 to July 2019	147	Adult	Not specified	Bivalirudin vs UFH	• ECMO duration<br>• Rates of thrombotic events<br>• Bleeding complications<br>• Transfusion requirements
Varnado et al, 2020	31st Annual ELSO Conference Abstracts, #182<br>ASAIO Journal, September 2020, Vol. 67, Supp. 3, ISSN 1058-2916	USA	Retrospective, single center	14	COVID-19 patients	VV	Bivalirudin vs UFH	• Time in TTR<br>• Bleeding & thrombotic complications
